# The fiber of persistent homology for trees

**DOI:** 10.1007/s41468-025-00213-z

**Published:** 2025-09-06

**Authors:** David Beers, Jacob Leygonie

**Affiliations:** Mathematical Institute, University of Oxford, Woodstock Road, Oxford, OX2 6GG UK

**Keywords:** Persistent homology, Merge trees, Inverse problems, Configuration spaces, 49N45, 55N99

## Abstract

Consider the space of continuous functions on a geometric tree *X* whose persistent homology gives rise to a finite generic barcode *D*. We show that there are exactly as many path connected components in this space as there are merge trees whose barcode is *D*. We find that each component is homotopy equivalent to a configuration space on *X* with specialised constraints encoded by the merge tree. For barcodes *D* with either one or two intervals, our method also allows us to compute the homotopy type of this space of functions.

## Introduction

### Motivation

Persistent Homology ($\textrm{PH}$) is a computable descriptor from Topological Data Analysis (TDA) which summarises complex geometric data. More precisely, the persistence map, denoted $\textrm{PH}$, takes as input a topological space *X* equipped with a real valued function $f:X\rightarrow \mathbb {R}$ and returns a collection $\textrm{PH}(f)=\{\textrm{PH}_i(f)\}_{i\ge 0}$ of *barcodes*, each of which is a multiset of intervals in the real line encoding the *i*-th dimensional topological variations across the sublevel sets of *f*. In a wide range of situations, persistent homology is robust to perturbations of the input data (Cohen-Steiner et al. 2007), which is one of the key reasons for its successful application to problems in data science, e.g. in neuroscience (Bendich et al. 2016), material sciences (Hiraoka et al. 2016), shape recognition (Li et al. 2014), and machine learning (Chen et al. 2019).

Complementarily, it is natural to ask how decisively $\textrm{PH}$ distinguishes distinct input functions *f*. Equivalently, we may ask which functions give rise to the same collection of barcodes $D=\{D_i\}_{i\ge 0}$. This inverse problem formally translates into studying the fiber $\textrm{PH}^{-1}(D)$ over a target *D*. The topological and geometric properties of $\textrm{PH}^{-1}(D)$ strongly depend on the underlying space *X* and on the space $\mathcal {F}$ of functions $f:X\rightarrow \mathbb {R}$ on which persistent homology is defined, which can be for instance the space of *filter functions* (or one of its subspaces) when *X* is a simplicial or CW complex, the space of Morse functions when *X* is a smooth manifold, or simply of continuous functions when *X* is merely a topological space.

For filter functions on a simplicial complex, it was observed in (Leygonie and Tillmann 2022) that $\textrm{PH}^{-1}(D)$ has the structure of a finite polyhedral complex. This polyhedral structure was exploited in (Leygonie and Henselman-Petrusek 2021) to design an algorithm for computing the homology groups of $\textrm{PH}^{-1}(D)$, and this algorithm was demonstrated on a menagerie of small examples. When $\mathcal {F}$ is the subspace of filter functions determined by their values on vertices, it was shown that every connected component of $\textrm{PH}^{-1}(D)$ is contractible when *X* is a simplicial decomposition of the unit interval (Cyranka et al. 2020), and homotopy equivalent to a circle when *X* is instead a simplicial decomposition of the circle (Mischaikow and Weibel 2021).

Cases where *X* is not a finite topological space have also been investigated. For *Morse-like* continuous functions on the unit interval, the number of path components of $\textrm{PH}^{-1}(D)$ was computed for generic barcodes (Curry 2018). For Morse functions on the 2-sphere $\mathbb {S}^2$ obtained by composing an embedding of $\mathbb {S}^2$ in $\mathbb {R}^3$ with the vertical projection, the tools developed in (Catanzaro et al. 2020) motivated conjectures on the number of connected components of $\textrm{PH}^{-1}(D)$. For general Morse functions on an arbitrary smooth compact manifold, it was proven in (Leygonie and Beers 2022) that the group of diffeomorphisms of *X* isotopic to the identity gives rise to an action on $\textrm{PH}^{-1}(D)$ which is transitive on each connected component. This allowed computing the homotopy type of path components of $\textrm{PH}^{-1}(D)$ for Morse functions on 1-dimensional and 2-dimensional oriented manifolds. Large families of point clouds in $\mathbb {R}^n$ with identical one-dimensional persistence under popular filtrations arising from point cloud data have also been identified (Smith and Kurlin 2022).

However, the tools developed in the above literature do not adapt easily to continuous functions on a topological space *X* that is not a manifold. In (Mischaikow and Weibel 2021), it was observed that when *X* is a star-like tree and the zero-dimensional barcode $D_0$ is the specific barcode that has only one finite interval, then the path connected components of $\textrm{PH}^{-1}(D)$ are wedges of circles. In this work, we analyse $\textrm{PH}^{-1}(D)$ in the case of an arbitrary generic barcode $D_0$, for continuous functions on the geometric realisation of an arbitrary tree.

The case of a tree is of particular interest as it is frequently encountered in applications of persistent homology to neuroscience, e.g. for analysing neuronal morphologies (Kanari et al. 2018, 2019) and brain functionalities (Bendich et al. 2016). In fact, a few other related inverse problems for topological descriptors on a tree have already been studied. For instance, statistical and algorithmic inverses of the Topological Morphology Descriptor (TMD) have been described in (Curry et al. 2021; Kanari et al. 2020). Another example is the study of the realisation problem for barcodes of functions on a tree, which have been investigated in (Johnson and Scoville 2022; Liu and Scoville 2020).

### Contributions and outline of contents

In this work we study the case when *X* is the geometric realisation of a tree ( hereafter we will refer to geometric realisations of trees as *geometric trees*), $\mathcal {F}$ is the space of continuous functions on *X*, and $D_0$ is a finite generic barcode. For this reason, *X* denotes any geometric tree for the remainder of the introduction. As we will observe in Section 6, for functions *f* on a geometric tree the barcodes $\textrm{PH}_i(f)$ for $i\ge 1$ are all empty, for any $f\in \mathcal {F}$. Therefore, to simplify notations, for the rest of the introduction (and in Section 6) we identify the set of barcodes $\textrm{PH}(f)=\{\textrm{PH}_i(f)\}_{i\ge 0}$ with $\textrm{PH}_0(f)$, the barcode of the zero dimensional persistent homology of *f*. Similarly, instead of using the notation *D* for the set of all barcodes $\{D_i\}_{i\ge 0}$, we identify the collection of barcodes *D* with $D_0$, the single non-empty barcode.

Our analysis relies upon the fact that for functions *f* on *X*, the persistence map factors in the following way:Here, the intermediate object $\textrm{MT}(f)$ is a topological space called the *merge tree* of *f*, which describes how the connected components of the sublevel sets $f^{-1}(-\infty ,t]$ appear and join together as *t* varies. Hence, to characterise the fiber of persistent homology in this setting, we can instead characterise the space of functions with a given merge tree and the space of merge trees that map to *D*. The main contributions of this work are:

- In Theorem 9, we provide sufficient conditions for a merge tree derived from a function *f* on a topological space to have a cellular merge tree structure.
- In Theorem 20, we show that if *T* is a cellular merge tree with *n* leaves then $\textrm{MT}^{-1}(T)$ is homotopy equivalent to a constrained version of the configuration space of *n* points on *X*, denoted $\textrm{Conf}(X,T)$, where the points must satisfy additional constraints determined by $T$.
- In Theorem 25, we show that $\textrm{Conf}(X,T)$, and hence $\textrm{MT}^{-1}(T)$, is path connected when *X* has a branch point. We deduce a 1-1 correspondence between path connected components in the fiber $\textrm{PH}^{-1}(D)$ and non-isomorphic merge trees with barcode *D*.
- We derive two important consequences of the above results for when *X* has at least one branch point: (i) in Corollary 36, we find a lower-bound on the distance between the path connected components in $\textrm{PH}^{-1}(D)$, and (ii) in Corollary 35, we count the number of such components using existing work on merge trees (Curry 2018; Kanari et al. 2020).

The paper is organised as follows.

In Section 2 we formally define the notions of trees, geometric trees, and merge trees. Additionally, we define the notion of a cellular merge tree, a merge tree equipped with a suitable cellular structure. We also formally define persistent homology, describe the relationship between the local minima of a function and its zero dimensional barcode, and detail how the persistence map factors for functions on geometric trees.

In Section 3, we show that a function on a compact connected Hausdorff space has a cellular merge tree if and only if it has finitely many local minima. Then we define the interleaving distance between merge trees and show that it is a true metric on the subspace of cellular merge trees. Section 4 is devoted to providing necessary and sufficient conditions for when a function on a geometric tree *X* has a given cellular merge tree.

In Section 5 we define the configuration space $\textrm{Conf}(X,T)$ and a few other intermediary configuration spaces constrained by rules determined by $T$. By a series of consecutive homotopy equivalences between these configuration spaces, the section culminates in a proof of Theorem 20, showing that $\textrm{MT}^{-1}(T)$, the space of continuous functions on *X* with merge tree $T$, is homotopy equivalent to $\textrm{Conf}(X,T)$.

Section 6 exploits this homotopy equivalence to deduce topological properties of $\textrm{MT}^{-1}(T)$ and $\textrm{PH}^{-1}(D)$ for generic barcodes *D*. The main result of this section, Theorem 25, says that $\textrm{Conf}(X,T)$ and hence $\textrm{MT}^{-1}(T)$ are connected when *X* has at least one branch point, i.e. *X* is not homeomorphic to an interval. This then allows us to provide a lower bound on the distance between any two path connected components in $\textrm{PH}^{-1}(D)$ (Corollary 35), which depends only on the barcode *D*. In addition, combining Theorem 25 with existing work enumerating the number of merge trees with a given barcode (Curry 2018; Kanari et al. 2020), we deduce in Corollary 36 that

$$\begin{aligned}\# \pi _0(\textrm{PH}^{-1}(D))=\prod _{[b,d)\in D} \# \big \{ [b',d') \in D \mid [b,d) \subset [b',d')\big \},\end{aligned}$$

whenever *X* has a branch point. We conclude by computing the homotopy type of $\textrm{PH}^{-1}(D)$ via $\textrm{Conf}(X,T)$ when *D* has either one or two intervals. When *D* has one interval, we deduce that $\textrm{PH}^{-1}(D)$ is contractible (Corollary 37). When *D* has two (overlapping) intervals, Corollary 38 shows that $\textrm{PH}^{-1}(D)$ is homotopic to a wedge of

$$\begin{aligned} -1 + \sum _{v\in N(X)} (\eta (v) - 1)(\eta (v) - 2) \end{aligned}$$

circles, where *N*(*X*) is the set of vertices in any triangulation of *X*, and $\eta (v)$ is the degree of vertex *v*.

## Background

### Trees, merge trees and cellular merge trees

A *tree* is a finite connected acyclic graph. It is *binary* if each vertex is the endpoint of at most 3 edges (Note we do not require a binary tree to have a root or to be ordered). The *geometric realisation* of a tree $T$ is a topological space given by a copy of the interval [0, 1] for each edge in $T$ with pairs of endpoints identified whenever they correspond to the same vertex of $T$. An *edge* in the geometric realisation of *T* is the image of an interval [0, 1] corresponding to an edge in *T*, while a *vertex* in the geometric realisation of *T* is the image of the endpoint of an interval corresponding to an edge *e* of *T*. An edge *e* is *incident* to a node *x* in a geometric realisation of a tree *T* if *x* is the image of an endpoint of the interval [0, 1] corresponding to *e*. The *degree* of a node in the geometric realisation of a tree is the number of incident edges it has. A node *x* is a *leaf* if it has degree 1. A node of degree three or greater is called a *branch point*. A *geometric tree* is the geometric realisation of a tree. After choosing a cellular decomposition via a tree *T*, a geometric tree is also equipped with a notion of edges, vertices, incident edges, degree, leaves, and branch points.

Between any two points on a tree it is well known that there is a unique non self-intersecting path. We refer to this path as the *shortest path* between two given points. Indeed any other path connecting a given two points contains the shortest path in its image. When $T$ is geometric, the discussion extends to disjoint closed connected nonempty subsets $A,B\subseteq T$: there is a unique shortest path $\textrm{ShortPath}(A,B)$ connecting them.

It follows that a subset $S\subseteq T$ of a geometric tree is path-connected if and only if it is connected. Namely, if *S* is not path connected, then the shortest path in *T* between *a* and *b* in *S* is not contained in *S*. Taking $U_1$ and $U_2$ to be the connected components of *T* minus a point in this shortest path, but not in *S*, we induce a disjoint open cover of *S*.

A rooted tree $(T,r)$ is a tree $T$ with a distinguished vertex *r*. A *leaf* in a rooted tree is a vertex not equal to *r* adjacent to exactly one other vertex. A *branch point* is a vertex adjacent to three or more vertices. If the root *r* is adjacent to two or more vertices, then we say that *r* is a branch point as well. A choice of root induces an orientation on the edges of any tree $T$ by the following procedure. We start by directing edges of $T$ adjacent to *r* away from *r*. Inductively, if an edge between *v* and $v'$ has not yet been oriented but an edge incident to *v* has been oriented, we orient the edge between *v* and $v'$ from *v* to $v'$. Whenever there is a directed edge from *v* to $v'$ we say that $v'$ is a *child* of *v*.

In a rooted tree say that a vertex $v'$ is a *descendant* of *v* and *v* is an *ancestor* of $v'$ if there is a directed path from *v* to $v'$, where potentially $v = v'$. For rooted trees, we denote by $\textrm{LCA}(v,v')$ the least common ancestor of *v* and $v'$. This is the vertex *u* ancestral to both *v* and $v'$ such that any other $u'$ an ancestor of both *v* and *v* is an ancestor of *u*.

Next, we introduce merge trees. We will make use of two distinct definitions of merge trees from the literature. In both cases, the merge tree is a topological space *X* equipped with a real-valued map $\pi :X\rightarrow \mathbb {R}$. This makes the following notion convenient:

#### Definition 1

A **function-filtered space** is a topological space *X* equipped with a continuous map $\pi :X\rightarrow \mathbb {R}$.

A **morphism** between two function-filtered spaces  $(X_1,\pi _1)$ and $(X_2,\pi _2)$ is a continuous map $\phi :X_1 \rightarrow X_2$ satisfying $\pi _1 = \pi _2 \circ \phi$. An **isomorphism** of function-filtered spaces is a morphism that is also a homeomorphism.

A continuous function $f:X\rightarrow \mathbb {R}$ yields a merge tree as defined in (Morozov et al. 2013), which is an instance of function-filtered space :

#### Definition 2

For a topological space *X* with a continuous function *f*, the associated **merge tree** $\textrm{MT}(f)$ is the quotient of the space

$$\begin{aligned} \textrm{epi}(f) := \{ (x,t)\in X\times \mathbb {R} :t \ge f(x) \} \end{aligned}$$

by the relation $(x,t) \sim (y,t)$ whenever *x* and *y* are in the same connected component of $f^{-1}(-\infty ,t]$.

**Fig. 1:**
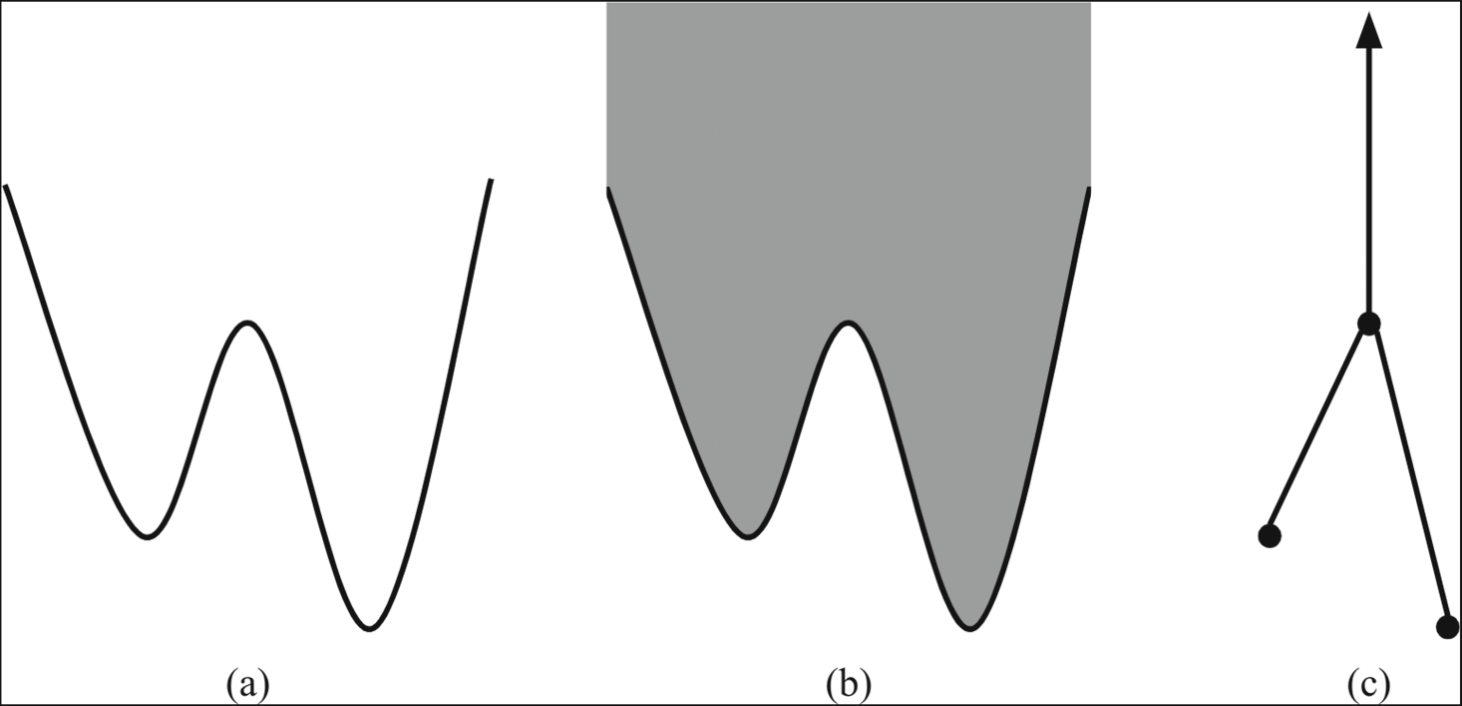
The construction of a merge tree from a function. (a) The graph of a function *f* on an interval. (b) Shaded is the content of $\textrm{epi}(f)$ strictly above the graph of *f*. (c) By sending connected components of horizontal slices to points we obtain $\textrm{MT}(f)$. It happens that this merge tree has a cellular structure, although in general this may not be the case

Since merge trees inherit a map $\pi _f$ to $\mathbb {R}$ from the second coordinate projection map on $\textrm{epi}(f)$, they are naturally viewed as function-filtered spaces. We illustrate the construction of a merge tree in Figure 1.

The other (non-equivalent) definition of merge trees that we will use appears for example in (Curry 2018). We provide an analogous definition here, along with the notion of a labeling motivated by, though slightly different from (Gasparovic et al. 2022).

#### Definition 3

A **cellular merge tree** $(T, \pi )$ is the quotient space of a geometric rooted tree $(T',r)$ and a half open interval:

$$\begin{aligned} T'\sqcup [0,1)/(r\sim 0), \end{aligned}$$

equipped with a real-valued continuous map $\pi :X \rightarrow \mathbb {R}$ satisfying

-  $\pi$ is strictly decreasing along edges in $T'$ oriented from the root *r*.
-  $\pi$ is strictly increasing to infinity along the half open interval [0, 1).

Given a cellular merge tree $(T,\pi )$, the map from $x \in \pi ^{-1}(t)$ to the connected component of $\pi ^{-1}(-\infty ,t]$ containing *x* is a bijection. Further, if there is a path from *x* to *y* in $T$ along which $\pi$ is increasing, then for $t \le s \le \pi (y)$, the connected component containing *x* in $\pi ^{-1}(-\infty , s ]$ is contained in the connected component containing *y* in $\pi ^{-1}(-\infty ,\pi (y)]$. Letting $f:=\pi$, it follows from this discussion that $(T,\pi ) = (T,f)$ is isomorphic to the function-filtered space $(\textrm{MT}(f),\pi _f)$. Hence we have an injection through which cellular merge trees form a subspace of regular merge trees (up to isomorphism).

For notational convenience we will sometimes refer to the subset (0, 1) of the half open interval of a cellular merge tree as $e_\infty$. For indexing convenience, we will often work with *labelled* cellular merge trees, for which an arbitrary ordering of the leaves $l_1,\cdots , l_n$ and of the nodes $v_1,\cdots ,v_m$ has been fixed. Note that we assume that these orderings do not allow repetitions of the leaves and of the nodes.

#### Definition 4

The **induced matrix** of a labelled cellular merge tree $(T,\pi )$ is given by

$$\begin{aligned} \mathcal {M}(T)_{ij} := \pi (\textrm{LCA}(l_i,l_j)). \end{aligned}$$

To simplify notations, when the context leaves it clear, we will write $\textrm{MT}(f)$ given a function *f* to designate the function-filtered space  $(\textrm{MT}(f),\pi _f)$, and similarly $T$ to designate the cellular merge tree $(T,\pi )$.

### Persistent homology

Fix a topological space *X* and a continuous function $f:X\rightarrow \mathbb {R}$. The function *f* gives rise to a collection of topological spaces $f^{-1}(-\infty ,t]$, nested by inclusion maps. Applying $i^\textrm{th}$ homology over a field $\mathbb {F}$ to the collection of spaces induces a collection of vector spaces indexed by $\mathbb {R}$, with linear maps induced by inclusion from the vector space indexed by *s* to the vector space indexed by *t* whenever $s\le t$. This collection  $\mathbb {V}_i(f)$ of vector spaces is called the $i^\textrm{th}$ *persistence module* *f*. The persistence modules *f* can also be thought of as functors from $(\mathbb {R}, \le )$ to the category of vector spaces. The following definition gives us a notion of well-behaved functions.

#### Definition 5

A persistence module $\mathbb {V}_i(f)$ is *pointwise finite dimensional* if $\dim H_i(f^{-1}(-\infty ,t])<\infty$ for all *t*. A function *f* is *pfd* if all its persistence modules $\mathbb {V}_i(f)$ are pointwise finite dimensional.

If $\mathbb {V}_i(f)$ is pointwise finite dimensional, i.e. $\dim H_i(f^{-1}(-\infty ,t])<\infty$ for all *t*, then the $i^{\text {th}}$ persistence module decomposes into a direct sum of modules (Crawley-Boevey 2015) indexed by a multiset $D_i$,

1 $$\begin{aligned} \mathbb {V}_i(f) \cong \bigoplus _{I\in D_i} M_I, \end{aligned}$$

where each $I\subseteq \mathbb {R}$ is an interval of the real line, and $M_I$ is defined to be the collection of vector spaces

$$\begin{aligned} M_I(t) = {\left\{ \begin{array}{ll} \mathbb {F} & t\in I \\ 0 & \text {else,} \end{array}\right. } \end{aligned}$$

with associated maps

$$\begin{aligned} M_I(s,t) = {\left\{ \begin{array}{ll} \textrm{id} & s,t\in I \\ 0 & \text {else.} \end{array}\right. } \end{aligned}$$

The collections of vector spaces $M_I$ are called *interval modules*.

The multiset of intervals $D_i$ is called the *barcode* in dimension *i* of *f*. We say that $D_i$ is *finite* if it is a finite collection of intervals.

When *f* is pfd, the collection of barcodes $\{\textrm{PH}_i(f)\}_{i\ge 0}$ associated to a function *f*, abbreviated $\textrm{PH}(f)$, is well-defined and referred to as its *persistent homology*.

### Persistent homology and local minima

In this section we show some relations between the zero dimensional barcode $\textrm{PH}_0(f)$ and the number of local minima of a function *f*. Throughout, we will use the following definition of a local minimum.

#### Definition 6

Given a topological space *X* and a map $f:X\rightarrow \mathbb {R}$, a nonempty subset $M\subseteq X$ is a **local minimum of** *f* if *M* is connected, *f* is constant on *M*, and any connected $M'$ strictly containing *M* also contains a point *x* satisfying $f(x) > f(M)$.

Note that if *f* is continuous then its local minima are each closed. This is true for the following reason. If *M* is a local minimum of a continuous function *f* with $f(M) = a$, then the closure $\overline{M}$ of *M* contains *M* and $f\big (\overline{M}\big ) = a$. Since the closure of a connected set is connected, *M* must be equal to its closure. Hence, if *X* is also compact, then the local minima of *f* are compact. In particular the local minima are compact when *f* is continuous and *X* is a geometric tree.

The above definition is a somewhat weak notion of a local minimum in the sense that it does not in general require that a local minimum *M* has a neighborhood *U* such that $f(U-M)>f(M)$. We use our definition of a local minimum here as it is necessary for our upcoming Theorem 9 to hold, as we will show in Remark 2.

#### Lemma 7

Let *X* be any topological space, $D_0$ be a finite barcode, and $f:X\rightarrow \mathbb {R}$ with $\textrm{PH}_0(f) = D_0$. Then *f* has finitely many local minima.

#### Proof

Let $M$ be a local minimum of *f*, and $m=f(M)$. Assume, seeking contradiction, that no interval of $D_0$ starts at *m*. Then we can find a range $[m-\epsilon , m]$ where no interval of $D_0$ starts. Using the decomposition (1) we see that the internal morphism $\mathbb {V}_0(f)(m-\epsilon )\rightarrow \mathbb {V}_0(f)(m)$ is surjective.

Note that $M\subseteq f^{-1}(-\infty ,m]$ is connected in *X* and hence is a disjoint union of path connected subspaces. Pick one of these subspaces $M_0$. Therefore there is a path-connected component $\Omega _{m-\epsilon }$ of $f^{-1}(-\infty ,m-\epsilon ]$ which lies in the same path-connected component as $M_0$ in $f^{-1}(-\infty ,m]$. Given a path $\gamma$ from $\Omega _{m-\epsilon }$ to $M_0$ in $f^{-1}(-\infty ,m]$, consider the set $M'=M\cup \textrm{im}\,\gamma$. This is the union of the set $\gamma$, which is path connected and hence connected, and the set *M*, which is also connected. Since $\textrm{im}\,\gamma$ and *M* intersect at an endpoint of $\gamma$ in $M_0\subseteq M$, the union $M \cup \textrm{im}\,\gamma$ is connected and contradicts that $M$ is a local minimum.

The same reasoning, working locally around local minima, shows that there are at least as many intervals in $D_0$ starting at *m* as there are local minima with value *m*. Since $D_0$ is finite, this implies that *f* has finitely many local minima. $\square$

The following result also ensures that the barcode of a continuous function on a tree with finitely many local minima is well-defined.

#### Lemma 8

Let *X* be a geometric tree and $f:X\rightarrow \mathbb {R}$ be a continuous function with finitely many local minima. Then *f* is pfd.

#### Proof

Let $t\in \mathbb {R}$. Let $\Omega$ be a path-connected component of $f^{-1}(-\infty ,t]$. Consider the following alternative:

1. Either *f* has constant value *t* over $\Omega$. Then $M:=\Omega$ is a local minimum of *f*, because any connected strict superset $M'$ will be included in some $f^{-1}(-\infty ,t']$ with $t'>t$, but not in $f^{-1}(-\infty ,t]$.
2. Or *f* attains a minimum $t'<t$ over one maximal connected subset $M\subseteq \Omega$. Then *M* is also a local minimum of *f* in the whole $X$.

In both cases, we can find a local minimum of *f* inside $\Omega$, and since *f* has finitely many local minima, we deduce that $f^{-1}(-\infty ,t]$ has finitely many path-connected components, i.e. $\dim H_0(f^{-1}(-\infty ,t])<+\infty$. This is because a subset of a geometric tree is connected if and only if it is path connected.

Finally, since *X* is a tree, its subsets are component-wise contractible, hence $\dim H_i(f^{-1}(-\infty ,t])=0$ for all $i>0$ and the result follows. $\square$

### The fiber of persistent homology on a tree

In this section we assume that $X$ is a geometric tree. Then all of its subsets are component-wise contractible, so its $i^\textrm{th}$ persistence modules are trivial for all $i>0$.

In this paper, we study the space of pfd continuous functions $f:X\rightarrow \mathbb {R}$ giving rise to a fixed barcode $D_0$:

$$\begin{aligned}\textrm{PH}_0^{-1}(D_0) := \bigg \{ f:X\rightarrow \mathbb {R}\text { pfd continuous} \mid \textrm{PH}_0(f)=D_0\bigg \}.\end{aligned}$$

We consider the topology on $\textrm{PH}_0^{-1}(D_0)$ induced by the supremum norm on continuous functions.

#### Remark 1

We can also consider this inverse problem more generally in the space of all continuous functions by working directly at the level of persistence modules: studying the space of continuous functions $$f:X\rightarrow \mathbb {R}$$ satisfying $$ \mathbb {V}_0(f) \cong \bigoplus _{I\in D_0} M_I$$. Our analysis could be conducted in this setting without substantial modifications, in particular because we will assume $$ D_0$$ to be finite. But to keep the exposition simple, this work assumes functions are pfd so that their barcodes are always defined.

As observed in (Morozov et al. 2013), the zero dimensional persistent homology of (*X*, *f*), for $f:X\rightarrow \mathbb {R}$ a pfd function, is also the zero dimensional persistent homology of $(\textrm{MT}(f),\pi _f)$. Indeed, the dimension of $H_0(f^{-1}(-\infty ,t])$ is exactly the number of path components of $f^{-1}(-\infty ,t]$, which, being a subset of a geometric tree, is the number of connected components of $f^{-1}(-\infty ,t]$. This is exactly the number of points in $\pi _{f}^{-1}(t)$. However, the set $\pi _f^{-1}(-\infty ,t]$ retracts onto $\pi _f^{-1}(t)$ via the homotopy

$$\begin{aligned} h_u: (x,s) \longmapsto (x,s(1-u) + tu). \end{aligned}$$

Hence for each *t* the map $x \mapsto (x,t)$ induces pointwise isomorphisms between $H_0(f^{-1}(-\infty ,t])$ and $H_0(\pi _f^{-1}(-\infty ,t])$. Further, these isomorphisms commute with inclusions arising from inequalities $s \le t$. Hence the persistence modules of (*X*, *f*) are completely determined by $\textrm{MT}(f)$. In other words the map $\textrm{PH}_0$ factors as a composite of mapsWe thus refer to the *persistent homology* on a merge tree as the second of these maps $\textrm{MT}(f) \mapsto \textrm{PH}_0(\pi _f)$, and its value on a given merge tree $\textrm{MT}(f)$ as the *barcode* of $\textrm{MT}(f)$. Note that, an isomorphism $\phi$ of function-filtered spaces acting on $\textrm{MT}(f)$ maps sublevel sets $\pi _f^{-1}(-\infty ,t]$ homeomorphically, and commutes with inclusion maps between sublevel sets. Therefore $\phi$ induces an isomorphism of persistence modules in dimension zero and so the barcode of $\textrm{MT}(f)$ is a function-filtered isomorphism invariant. Viewing cellular merge trees as a subclass of merge trees, we then have that the barcode of a cellular merge tree of $(T,\pi )$ is $\textrm{PH}(\pi )$, which is also function-filtered isomorphism invariant.

These observations naturally organise the problem of computing the fiber $\textrm{PH}_0^{-1}(D_0)$ into two consecutive steps: we will first study which functions have a given merge tree, and second, which merge trees have a given barcode.

## Tree structure and metric for merge trees

### When Merge trees are trees

It is tempting to assume that $(\textrm{MT}(f),\pi _f)$ is always a cellular merge tree, however this is not the case, even when *X* is very simple.

#### Example

If $X = (-\infty , 0]$ and $f(x) = e^x$, $\textrm{MT}(f)$ is an interval with two open endpoints. This cannot be a cellular merge tree since cellular merge trees have at most one open endpoint.

The following result gives conditions under which $\textrm{MT}(f)$ is indeed a cellular merge tree.

#### Theorem 9

Let *X* be a compact connected Hausdorff space and $f:X \rightarrow \mathbb {R}$ be a continuous function. Then $(\textrm{MT}(f),\pi _f)$ is a cellular merge tree if and only if *f* has finitely many local minima. Moreover, $(\textrm{MT}(f),\pi _f)$ is isomorphic to the labelled cellular merge tree $(T, \pi )$ with leaves $l_1,\ldots ,l_n$ such that

1. $$\pi (l_i) = m_i$$ for $$1\le i \le n$$
2. $$\mathcal {M}(T)_{ij} = t_{ij}$$

if and only if the following conditions on *f* are satisfied:

1. The function *f* has *n* local minima $X_1,\cdots ,X_n$, with $f(X_i) = m_i$ for $1\le i \le n$.
2. For any $1\le i<j \le n$, the infimum of values *t* where $X_i$ and $X_j$ are in the same connected component of $f^{-1}(-\infty ,t]$ is $t_{ij}$, i.e. $t_{ij}=\inf \{t \mid (X_i,t)\sim (X_j,t)\}$.

We break the proof up into a series of lemmas, the first two of which will help with the ‘if’ direction. For the following two lemmas we fix *X* to be compact, connected, and Hausdorff, and a function *f* with finitely many local minima satisfying the last two conditions in the theorem.

#### Lemma 10

Let (*y*, *t*) be a representative of an element in the quotient set $\textrm{MT}(f)$. There exists a local minimum $M$ of *f* such that (*x*, *t*) is a representative of the same element as  (*y*, *t*) for any *x* in $M$. In particular, $M$ can be chosen such that $f(M) \le f(y)$. As a result, for any *t*, there are at most as many connected components of $f^{-1}(-\infty ,t]$ as local minima of *f*.

#### Proof

Fix *y* and *t*. Let $\Omega _y$ denote the connected component of *y* in $f^{-1}(-\infty ,t]$. The set $\Omega _y$ is a closed subset of a compact set, and therefore is compact. Let $m:=\min f_{|\Omega _y}$, and let $M\subseteq \Omega _y$ be a connected component of $f^{-1}(m)\cap \Omega _y$. We claim $M$ is a local minimum of *f* in *X*.

Suppose $M'\subseteq X$ is a connected set containing $M$ on which *f* is never greater than *m*. Since $M'\subseteq f^{-1}(-\infty ,m] \subseteq f^{-1}(-\infty ,t]$, we have $M'\subseteq \Omega _y$, and therefore $M'\subseteq f^{-1}(m)$ because $m=\min f_{|\Omega _y}$. Thus $M=M'$. It follows that $M$ is a local minimum of *f* in *X*. Meanwhile, since $M$ minimises *f* on $\Omega _y$, it must be the case that $f(M) \le f(y)$.

To each connected component $\Omega _y\subseteq f^{-1}(-\infty ,t]$, we associate one of its local minima $M\subseteq \Omega _y$, and the last part of the lemma follows. $\square$

#### Lemma 11

For any $1\le i<j \le n$, local minima $X_i$ and $X_j$ are connected in $f^{-1}(-\infty ,t_{ij}]$.

#### Proof

Suppose the opposite. Let $\Omega _i$ be the connected component of $X_i$ in $f^{-1}(-\infty ,t_{ij}]$. By Lemma 10 there are finitely many connected components in $f^{-1}(-\infty ,t_{ij}]$, so $\Omega _i$ is both open and closed in $f^{-1}(-\infty ,t_{ij}]$. Thus $\Omega _i$ and ${\Omega }_j:=f^{-1}(-\infty ,t_{ij}]-\Omega _i$ are disjoint sets in *X* that are both open and closed in $f^{-1}(-\infty ,t_{ij}]$. In particular, ${\Omega }_j$ is nonempty as it contains $X_j$. Since $\Omega _i$ and ${\Omega }_j$ are each closed, disjoint, and nonempty in a closed subspace, they are closed, disjoint, and nonempty subsets of *X*. Since *X* is compact and Hausdorff and hence normal, there exists disjoint open sets $U_i\supseteq \Omega _i$ and $U_j\supseteq {\Omega }_j$ in *X*. Therefore $X - U_i\cup U_j$ is closed and thus compact in *X*. If $X-U_i\cup U_j$ is empty, this would imply $X =U_i\cup U_j$ is disconnected, contradicting the hypotheses of the theorem. Hence $X-U_i\cup U_j$ is nonempty and compact and we may let $m:=\min f|_{X - U_i\cup U_j}$.

The value $m$ is greater than $t_{ij}$ since $f^{-1}(-\infty ,t_{ij}]$ is contained in $\Omega _i\cup {\Omega }_j$. Therefore by restricting the open cover consisting of $U_i$ and $U_j$ to $f^{-1}(-\infty ,t]$ for any $t<m$, we see that $\Omega _i$ and ${\Omega }_j$ lie in separate connected components of $f^{-1}(-\infty ,t]$. In particular, since $m>t_{ij}$ we may take $t>t_{ij}$, contradicting the hypotheses of the theorem. $\square$

We will also need the following lemma for the ‘only if’ direction of the theorem.

#### Lemma 12

Let *X* be an arbitrary topological space. Suppose $f:X \rightarrow \mathbb {R}$ is a continuous map such that $\textrm{MT}(f)$ is isomorphic to a cellular merge tree $(T,\pi )$, with isomorphism $\phi :T\rightarrow \textrm{MT}(f)$. A subset $M\subseteq X$ is a local minimum of *f* if and only if there exists a leaf *l* of *T* such that

2 $$\begin{aligned} M = \{x \in X: (x,\pi (l)) \text { is a representative of }\phi (l)\in \textrm{MT}(f)\}. \end{aligned}$$

It follows that *f* has exactly as many local minima as *T* has leaves.

#### Proof

We first prove the “if” portion of the lemma. Suppose *M* is as in Equation (2). Thus, by the definition of $\textrm{MT}(f)$, *M* is a connected component of $f^{-1}(-\infty ,\pi (l)]$, so *M* is a connected subset of *X*. Also from the definition of *M* it is immediate that for all $x\in M$, $f(x)\le \pi (l)$. If there existed a point $x\in M$ such that $f(x)<\pi (l)$, then we would have the collection of representatives (*x*, *t*) for $f(x) \le t \le \pi (l)$ defining a path in *T* to $\phi (l)$ on which $\pi _f$ is strictly increasing. This contradicts that $\textrm{MT}(f)$ is isomorphic to *T*, since *l* is a leaf, so in fact *f* is constant on *M*. Finally, suppose $M'$ is a connected subset of *X* that strictly contains *M*. Let $\Gamma (M')\subseteq \textrm{epi}(f)$ denote the graph of *f* on $M'$, i.e.

$$\begin{aligned} \Gamma (M') := \{(x,t)\in X\times \mathbb {R}: x \in M' \text { and }f(x) = t\}. \end{aligned}$$

We let $\textrm{MT}(M')$ denote the image of $\Gamma (M')$ in the quotient space $\textrm{MT}(f)$ defined taking (*x*, *t*) to the element it represents. The space $\Gamma (M')$ is connected and the quotient map $\Gamma (M') \rightarrow \textrm{MT}(M')$ is continuous, so $\textrm{MT}(M')$ is connected. Since $M \subseteq M'$, $\phi (l) \in \textrm{MT}(M')$. However, it cannot be the case that $\textrm{MT}(M') = \{\phi (l)\}$, as this would imply that $M'\subseteq M$. Therefore, being connected, $\textrm{MT}(M')$ must intersect the interior of the edge in $\textrm{MT}(f)$ incident to $\phi (l)$. Hence there must exist a point $y\in M'$ such that $f(y) > \pi (l)$. This proves that *M* is indeed a local minimum.

For the “only if” direction, suppose *M* is a local minimum of *f*. Using the notation $\textrm{MT}(M)$ as in the first half of the proof, we have by the same argument used in the first half of the proof that $\textrm{MT}(M)$ is connected since *M* is connected. Moreover, since *f* is constant on *M*, $\pi _f$ is constant on $\textrm{MT}(M)$. These facts imply that $\textrm{MT}(M)$ is a single point $\{\phi (p)\} \subseteq \textrm{MT}(f)$. This is because level sets of $\pi _f$ are finite collections of points as $\textrm{MT}(f)$ is isomorphic to *T*. Now define

$$\begin{aligned} M' := \{x \in X: (x,\pi (p)) \text { is a representative of }\phi (p)\in \textrm{MT}(f)\}. \end{aligned}$$

It follows from the definition of $M'$ that $f(x) \le \pi (p)$ for all $x\in M'$ and moreover that $M'$ is a connected component of $f^{-1}(-\infty ,\pi (p)]$, and hence is connected in *X*.

If *p* is not a leaf, then there is a path from some $q\in T$ to *p* along which $\pi$ is increasing. Let $(x,\pi (q))$ denote a representative of $\phi (q)$. We thus have that $(x,\pi (p))$ is a representative of $\phi (p)$, and yet $f(x) \le \pi (q) < \pi (p)$. This means that $x \in M'$ but $x\notin M$. Since *M* is connected, *f* is bounded above by $\pi (p) = f(M)$ on $M'$, and $M'$ strictly contains *M*, *M* cannot be a local minimum. Thus by contradiction we have shown that *p* must be some leaf *l* of *T*. The set $M'$ is still connected, satisfies that $f(x) \le \pi (p) = \pi (l)$ for $x\in M'$, and contains *M*. Hence *M* is not a local minimum unless the containment $M\subseteq M'$ is not strict, i.e. $M=M'$. This proves the other direction of the lemma. $\square$

We next turn to the heart of the proof and find the actual tree structure of the merge tree $\textrm{MT}(f)$.

#### Proof of Theorem 9

Let *f* satisfy the last pair of conditions in the theorem. Let $x_1,\ldots ,x_n$ be points in each of the local minima in *X* achieving the values $m_1,\cdots ,m_n$ under *f*, and let:

$$\begin{aligned}T:= \bigg ( \bigsqcup _{i=1}^n \{x_i\}\times [m_i,+\infty ) \bigg )/ \bigg \{ (x_i,t)\sim (x_j,t) \mid t\ge t_{ij} \bigg \}. \end{aligned}$$

So $T$ is a disjoint union of the *n* right-open intervals $\{x_i\}\times [m_i,+\infty )$ with the *i*-th interval identified to the *j*-th interval at and beyond the threshold $t_{ij}$. In particular, $T$ has the structure of a cellular merge tree satisfying the first pair of conditions of the theorem.

We have a continuous map $T \rightarrow \textrm{MT}(f)$ given by $(x_i,t) \mapsto (x_i,t)$. This map is is well-defined by Lemma 11. We also have a continuous map in the other direction, namely $(x,t)\mapsto (x_i,t)$ where $x_i$ is provided by Lemma 10 to ensure $(x,t)\sim (x_i,t)$ in $\textrm{epi}(f)$ (recall Definition 2). Therefore $T$ and $\textrm{MT}(f)$ are isomorphic. This proves the “if” part of both statements in the theorem.

For the “only if” part of the theorem, suppose *f* has a cellular merge tree $(T,\pi )$, with leaves $l_i$ for $1\le i\le n$. Define $m_i= \pi (l_i)$ and $t_{ij}=\mathcal {M}(T)_{ij}$. By Lemma 12, *f* has *n* local minima $X_1,\ldots , X_n$. Let $s_i = f(M_i)$ and $s_{ij} = \inf \{t \mid (X_i,t)\sim (X_j,t)\}$. By the part of the theorem we have already proven, $\textrm{MT}(f)$ is isomorphic to a cellular merge tree $(T',\pi ')$ with *n* leaves $l_1',\ldots ,l_n'$ with $\pi '(l_i') = s_i$ and $\mathcal {M}(T')_{ij} = s_{ij}$ for $i\ne j$.

The merge trees $(T,\pi )$ and $(T',\pi ')$ are both isomorphic to $\textrm{MT}(f)$. Let $h:T \rightarrow T'$ be an isomorphism of merge trees, which must send leaves to leaves, being a homeomorphism. After potentially re-indexing the $X_i$ (and hence the $l_i'$) we have that *h* sends leaf $l_i$ to leaf $l_i'$ for each *i*. After this reordering, the map *h* is an isomorphism of $(T,\pi )$ and $(T',\pi )$ when viewed as labelled merge trees on *n* leaves. Thus, by (Munch and Stefanou 2019, Corollary 4.3)^1^, $\mathcal {M}(T) = \mathcal {M}(T')$. Equality of diagonal entries implies that $\pi (l_i) = \pi '(l_i')$ for each *i*, whereas equality of off-diagonal entries implies $s_{ij} = t_{ij}$ for all $i\ne j$. This concludes the proof. $\square$

#### Remark 2

If we defined merge trees using the equivalence relation $(x,t) \sim (y,t)$ whenever *x* and *y* are in the same path component of $f^{-1}(-\infty ,t]$ instead of the same connected component, Theorem 9 would not hold. For a counterexample, consider the so-called topologist’s sine curve

$$\begin{aligned} S = \{(0,t)\in \mathbb {R}^2 : t\in [-1,1]\}\cup \{(x,\sin \frac{1}{x})\in \mathbb {R}^2:x\in (0,1]\}. \end{aligned}$$

It is well known that *S* is closed and connected but has two path components. Taking *B* to be a closed disk covering *S* and $f:B\rightarrow \mathbb {R}$ the Euclidean distance from *S*, we see that $f^{-1}(-\infty ,0]=S$ is not path-connected. Thus $\textrm{MT}(f)$ cannot be a cellular merge tree as it is not Hausdorff: any neighborhood of either point *p* with $\pi _f(p)=0$ contains both such points.

Moreover, suppose we instead defined a local minimum of *f* as a connected closed set *M* such that *f* is constant on *M* and such that there exists a neighborhood *U* of *M* satisfying $f(U-M) > f(M)$. While this might seem to be a more sensible definition of a local minimum, Theorem 9 does not hold with this definition either. For a counterexample, take $X = [0,1]$ and $C \subseteq X$ the Cantor middle-thirds set. Define *f*(*x*) to be the distance of *x* to *C*. Since *C* is closed, $f^{-1}(0) = C$. Due to the piecewise linear nature of *f* on connected components of $X-C$, it is easily checked that no local minimum of *f* exists which intersects $X-C$. Since *C* is totally disconnected, this means any local minimum of *f* is a point in *C*. However, since *C* has no isolated points, every neighborhood *U* of each $x\in C$ intersects *C* somewhere else. Therefore $U-\{x\}$ contains a point *y* with $f(y) = f(x)$, so *x* is not a local minimum. So in fact *f* has no local minima.

This would produce a contradiction of the theorem since $\pi _f^{-1}(0)$ has a point for each element in *C*, and hence is uncountable, which is impossible for a cellular merge tree. Of course, using the definition of a local minimum taken in this paper one can check that *f* has infinitely local minima and so does not contradict Theorem 9.

### Interleaving distance on cellular merge trees

In this section we analyse the interleaving distance, a pseudo-distance on merge trees (Morozov et al. 2013, Lemma 1). We show that it is in fact a genuine distance on the subspace of cellular merge trees (up to isomorphism).

Aside from the map $\pi _f$, merge trees also come equipped with $\epsilon$-*shift* maps, for $\epsilon {\ge } 0$:

$$\begin{aligned}i_f^\epsilon :(x,t)\in \textrm{MT}(f) \longmapsto (x,t+\epsilon )\in \textrm{MT}(f). \,\end{aligned}$$

We will often omit subscripts, writing $i_f^\epsilon$ as $i^\epsilon$.

#### Definition 13

Let *f* and *g* be two continuous functions on *X*. An $\epsilon$-**interleaving** between $\textrm{MT}(f)$ and $\textrm{MT}(g)$ is a pair of continuous functions $\alpha ^\epsilon :\textrm{MT}(f) \rightarrow \textrm{MT}(g)$, $\beta ^\epsilon : \textrm{MT}(g) \rightarrow \textrm{MT}(f)$ satisfying the following equations

$$\begin{aligned}&\beta ^\epsilon \circ \alpha ^\epsilon = i^{2\epsilon } \qquad \pi _g(\alpha ^\epsilon (x)) = \pi _f(x) + \epsilon \\&\alpha ^\epsilon \circ \beta ^\epsilon = i^{2\epsilon } \qquad \pi _f(\beta ^\epsilon (y)) = \pi _g(y) + \epsilon . \end{aligned}$$

The **interleaving distance** $d_I(\textrm{MT}(f),\textrm{MT}(g))$ is defined as the infimum of values $\epsilon$ such that $\textrm{MT}(f)$ and $\textrm{MT}(g)$ are $\epsilon$-interleaved.

**Fig. 2:**
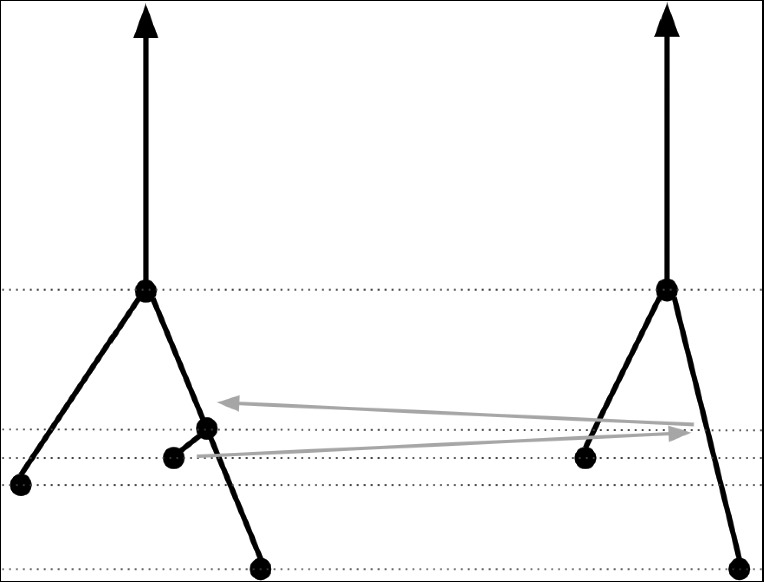
An interleaving between two merge trees

We illustrate an interleaving between two merge trees in Figure 2.

The following result is immediate from the study of cellular merge trees carried out in (Gasparovic et al. 2022), but surprisingly is not stated there, so we provide a quick proof using the results of (Gasparovic et al. 2022) for the sake of completeness.

#### Proposition 14

The interleaving distance is a metric on the subspace of cellular merge trees (up to isomorphism).

#### Proof

By (Morozov et al. 2013, Lemma 1) the interleaving distance is an extended pseudometric on cellular merge trees, so it remains to show that the interleaving distance is real valued on cellular merge trees and that two cellular merge trees $(T_1, \pi _1)$ and $(T_2,\pi _2)$ are isomorphic if they have interleaving distance zero.

Fix any two cellular merge trees $(T_1, \pi _1)$ and $(T_2,\pi _2)$. Corollary 4.4 of (Gasparovic et al. 2022) (which in turn depends on (Touli and Wang 2018, Theorem 1)), implies that $d_I$ is real-valued. Moreover if $d_I(T_1,T_2)=0$, (Gasparovic et al. 2022, Corollary 4.4) (see also their Definition 2.11) implies that there is a continuous map $\alpha :T_1\rightarrow T_2$ satisfying

1. If $\alpha (x) = i^\epsilon \alpha (y)$ then there is some $\delta$ such that $x = i^\delta y$.
2. $$\alpha $$ is surjective.
3. $$\pi _1(x) = \pi _2(\alpha (x))$$

Properties 1 and 3 together imply that if $\alpha (x) = i^\epsilon \alpha (y)$ then $x = i^\epsilon y$. Taking $\epsilon = 0$ we see that if $\alpha (x) = \alpha (y)$, then *x* = *y*, so $\alpha$ is injective. Therefore $\alpha$ is a bijection. Hence the property that $\alpha (x) = i^\epsilon \alpha (y) \implies x = i^\epsilon y$ shows that $\alpha ^{-1}$ is continuous. Therefore $\alpha$ is a merge tree isomorphism, completing the proof. $\square$

#### Proposition 15

Let *X* be a geometric tree, and let $f,g:X\rightarrow \mathbb {R}$ be continuous pfd functions with the same finite barcode $D_0$ in dimension zero. There is a positive number $\delta$ such that if

$$\begin{aligned} \Vert f-g\Vert _\infty < \delta , \end{aligned}$$

then $\textrm{MT}(f)$ and $\textrm{MT}(g)$ are isomorphic.

#### Proof

By Lemma 7 and Theorem 9, $\textrm{MT}(f)$ and $\textrm{MT}(g)$ are both cellular. By Curry (2018) there are only finitely many isomorphism classes of cellular merge trees $T_1,\ldots , T_n$ whose persistent homology is $D_0$. Set $\delta = \min _{i\ne j} d_I(T_i,T_j)$. This value is nonzero since it is a minimum of finitely many values, all of which are nonzero by Proposition 14. Since the persistence map factors through the merge tree operation for geometric trees *X*, we have that, up to isomorphism, $\textrm{MT}(f) = T_k$ and $\textrm{MT}(g) = T_l$ for some *k* and *l*. The stability theorem for the interleaving distance (Morozov et al. 2013, Theorem 2) gives us that

$$\begin{aligned} d_I(T_k,T_l) = d_I(\textrm{MT}(f),\textrm{MT}(g)) \le \Vert f-g\Vert _\infty < \delta = \min _{i\ne j} d_I(T_i,T_j), \end{aligned}$$

implying that $k = l$, and so $\textrm{MT}(f)$ and $\textrm{MT}(g)$ are isomorphic. $\square$

If we let $\delta _L$ be the minimum distance between pairs of non-equal interval left endpoints in $D_0$ and $\delta _R$ be the minimum distance between pairs of non-equal right endpoints, we conjecture that the largest value $\delta$ satisfying the conclusion of the previous proposition is $\min (\delta _L, \delta _R)$. We moreover conjecture that the same is true when *X* is any compact Hausdorff space.

## Functions on a tree with a given merge tree

In this section, we let $X$ be a geometric tree. In this context, the following is a convenient reformulation of Theorem 9. Recall that $\textrm{ShortPath}(X_i,X_j)$ denotes the shortest path in *X* joining two closed sets $X_i$ and $X_j$

### Proposition 16

Let $f:X\rightarrow \mathbb {R}$ be a continuous function with finitely many local minima. Then $\textrm{MT}(f)$ is isomorphic to a cellular merge tree $T$ with leaves $l_1,\ldots ,l_n$ satisfying $\pi (l_i) = m_i$ if and only if both the following conditions are satisfied:

1. The function *f* has *n* local minima $X_1,\cdots ,X_n$ with values $m_1,\cdots ,m_n$.
2. For any $i\ne j$, the maximum of *f* restricted on $\textrm{ShortPath}(X_i,X_j)$ equals $\mathcal {M}(T)_{ij}$.

### Proof

By Theorem 9, $\textrm{MT}(f)$ is a cellular merge tree, and it is isomorphic to $T$ if and only if:

1. The function *f* has *n* local minima $X_1,\cdots ,X_n$ with values $m_1,\cdots ,m_n$.
2. For any $1\le i<j \le n$, we have $\mathcal {M}(T)_{ij}=\inf \{t \mid (X_i,t)\sim (X_j,t)\}$.

Furthermore by Lemma 11 we can replace the infimum by a minimum in the second condition. Since *X* is a geometric tree it is then clear that

$$\begin{aligned} \min \{t\mid X_i \text { and } X_j \text { are connected in } f^{-1}(-\infty ,t]\}= \max f_{|\textrm{ShortPath}(X_i,X_j) } \end{aligned}$$

$$\square $$

For the next result, we will restrict our attention to cellular merge trees whose branch points and leaf values are non-degenerate.

### Definition 17

A cellular merge tree $(T,\pi )$ is *generic* if it is a binary tree (each internal node has two children), and all leaves have distinct projection values.

If a generic cellular merge tree $(T,\pi )$ has *n* leaves then it has $(n-1)$ internal nodes. For the rest of the section we fix a generic labelled cellular merge tree $T$, and fix a cellular structure on $T$ such that each internal node is a branch point. For each internal node $v\in T$, we fix an arbitrary labelling $Lv$ and $Rv$ of its two children.

The nodes of a cellular merge tree $T$ are endowed with a partial order $\preceq$ where $v\preceq v'$ whenever $v$ is a descendant of $v'$. In particular, for generic cellular merge trees $Lv\preceq v$ and likewise $Rv\preceq v$. For $1\le i \le n$ we denote by $m_i:=\pi (l_i)$ the value of a leaf.

Now we make use of the fact that cellular merge trees $T$ have distinct projection values on their leaves. If $X_1,\ldots ,X_n$ are the local minima of a function *f* with merge tree $T$, then we have the following two bijections:

$$\begin{aligned} f&:\{X_1,\ldots , X_n\} \rightarrow \{m_1,\ldots ,m_n\}\\ \pi&:\{l_1,\ldots ,l_n\} \rightarrow \{m_1,\ldots ,m_n\}, \end{aligned}$$

where the first bijection is guaranteed by Proposition 16 (or Theorem 9). As a result we have a canonical bijection between the local minima of *f* and the leaves of $T$. We always label local minima so that $X_i$ is mapped to $l_i$ by this bijection (and hence *f* maps $X_i$ to $m_i$). Note this bijection would not be canonical if two of the $m_i$ shared the same value.

Now we arrive at our most useful characterisation of functions with a given merge tree, expressed in terms of shortest paths and convex hulls of local minima $X_i$ of the function *f*. We define the *convex hull* of a collection $\mathcal {C}$ of closed subsets, denoted $\textrm{Conv}(\mathcal {C})$, as the union of points on shortest paths between elements of (potentially identical) sets in $\mathcal {C}$:

$$\begin{aligned} \textrm{Conv}(\mathcal {C}):= \bigcup _{A,B \in \mathcal {C}}\;\bigcup _{\begin{array}{c} a\in A\\ b\in B \end{array}}\textrm{ShortPath}(a,b).\end{aligned}$$

Clearly, convex hulls are connected because *X* is connected.

### Proposition 18

Let *X* be a geometric tree and $f:X\rightarrow \mathbb {R}$ be a continuous function with finitely many local minima. Then $\textrm{MT}(f)$ is isomorphic to a generic cellular merge tree $T$ with leaves $l_1,\ldots ,l_n$ satisfying $\pi (l_i) = m_i$ if and only if both the following conditions are satisfied:

1. The function *f* has *n* local minima $X_1,\cdots ,X_n$ with values $m_1,\cdots ,m_n$.
2. Given a node $v$ (possibly a leaf), let $X_f = (X_1,\ldots ,X_n)$ be the collection of local minima of *f*, and
  $$\begin{aligned}\textrm{Conv}_{X_f}(v):=\textrm{Conv}\bigg \{X_i \mid \text { leaf } l_i \text { is a descendant of } v\text { in }\textrm{MT}(f) \bigg \}\subseteq X.\end{aligned}$$
  Then, for any $v$ among the internal nodes $v_1,\ldots ,v_{n-1}$ of $T$, we have:
  3 $$\begin{aligned} \max \big \{f(x) \mid x \in \textrm{ShortPath}(\textrm{Conv}_{X_f}(Lv),\textrm{Conv}_{X_f}(Rv))\big \}=\pi (v), \end{aligned}$$
  and the maximum is attained at a unique connected subset $Y_k$ of the shortest path.

We refer to each set $Y_k$ as a *saddle* of *f*. For convenience, we define

$$\begin{aligned} \textrm{ShortPath}_{X_f}(v) := \textrm{ShortPath}(\textrm{Conv}_{X_f}(Lv),\textrm{Conv}_{X_f}(Rv)). \end{aligned}$$

### Proof

Let *f* be a continuous function with finitely many local minima $X_1,\cdots ,X_n$ with values $m_1,\cdots ,m_n$. This is condition 1 of both Proposition 18 and Proposition 16. Therefore, we are left to show that condition 2 of Proposition 18 holds if and only if condition 2 of Proposition 16 holds.

$(\Rightarrow )$ We show by induction on nodes $v$, sorted by increasing $\pi$-values and breaking ties arbitrarily, that the maximum value of *f* on the convex hull $\textrm{Conv}_{X_f}(v)$ is $\pi (v)$. Note that this is true for any leaf $l_i$ since $f(X_i)=m_i=\pi (l_i)$. Now let $v\in T$ be an internal node, such that *f* on $\textrm{Conv}_{X_f}(Lv)$ has maximum $\pi (Lv)$ and on $\textrm{Conv}_{X_f}(Rv)$ has maximum $\pi (Rv)$. Since *f* on $\textrm{ShortPath}_{X_f}(v)$ has maximum value $\pi (v)$, and $\pi (v)\ge \pi (Lv)$ as well as $\pi (v)\ge \pi (Rv)$, we deduce that the maximum value of *f* on

$$\begin{aligned} \textrm{Conv}_{X_f}(v)= \textrm{Conv}_{X_f}(Lv) \cup \textrm{Conv}_{X_f}(Rv)\cup \textrm{ShortPath}_{X_f}(v) \end{aligned}$$

is $\pi (v)$, which completes the induction.

Let $X_i$ and $X_j$ be two local minima, $i\ne j$, as in condition 2 of Proposition 16. Let $v$ be the least common ancestor of $l_i$ and $l_j$. We must have either $l_i \preceq Lv$ and $l_j \preceq Rv$, or $l_j \preceq Lv$ and $l_i \preceq Rv$. We assume the first situation holds without loss of generality. Then, $X_i$ is a subset of $\textrm{Conv}_{X_f}(Lv)$ and $X_j$ is a subset of $\textrm{Conv}_{X_f}(Rv)$, hence $\textrm{ShortPath}_{X_f}(v)$ is a subset of $\textrm{ShortPath}(X_i,X_j)$.

Therefore, on the one hand the maximum *M* of *f* over $\textrm{ShortPath}(X_i,X_j)$ is at least its maximum over $\textrm{ShortPath}_{X_f}(v)$, which is $\pi (v)$. On the other hand, since $\textrm{ShortPath}(X_i,X_j)$ is a subset of $\textrm{Conv}_{X_f}(v)$, *M* is also at most $\pi (v)$ by our earlier inductive argument. Thus $M=\pi (v)$, and condition 2 of Proposition 16 is proved.

$(\Leftarrow )$ We assume that condition 2 in Proposition 16 holds, which is equivalent to

$$\begin{aligned} \text {for any internal node } v\in T, \forall l_i\preceq Lv\text { and } l_j \preceq Rv, \, \max f_{|\textrm{ShortPath}(X_i,X_j) }= \pi (v). \end{aligned}$$

Another induction argument shows that the maximum value of *f* on the convex hull $\textrm{Conv}_{X_f}(v)$ equals $\pi (v)$ for any node $v$ and is attained on $\textrm{ShortPath}_{X_f}(v)$ for any internal node $v\in T$. As a base case, this is clear when $v$ is a leaf $l_i$, since $f(X_i) = m_i = \pi (l_i)$. Now take $v$ to be an internal node and assume the statement has already been proven for $Rv$ and $Lv$. Pick a leaf $l_i\preceq Lv$ and another leaf $l_j \preceq Rv$. Since $X_i \subseteq \textrm{Conv}_{X_f}(Lv)$ and $X_j \subseteq \textrm{Conv}_{X_f}(Rv)$ we have that

$$\begin{aligned} \textrm{Conv}_{X_f}(v)&= \textrm{Conv}_{X_f}(Lv) \cup \textrm{Conv}_{X_f}(Rv)\cup \textrm{ShortPath}_{X_f}(v)\\&=\textrm{Conv}_{X_f}(Lv) \cup \textrm{Conv}_{X_f}(Rv)\cup \textrm{ShortPath}(X_i,X_j). \end{aligned}$$

By the above equation, the maximum of *f* on $\textrm{Conv}_{X_f}(v)$ is $\pi (v)$, since the maximum of *f* on $\textrm{ShortPath}(X_i,X_j)$ is $\pi (v)$ and $\pi (v) \ge \pi (Rv),\pi (Lv)$. Then by the first line of the above equation, the maximum of *f* must be attained on $\textrm{ShortPath}_{X_f}(v)$, since $\pi (v) \ge \pi (Rv),\pi (Lv)$. This completes the induction.

Now let $v$ be an internal node. We are left to show that the maximum value $\pi (v)$ of *f* on $\textrm{Conv}_{X_f}(v)$ is attained at a unique connected subset of the shortest path. Assume, seeking contradiction, that the maximum $\pi (v)$ is attained at a disconnected subset $Y_k$ of the shortest path $\textrm{ShortPath}_{X_f}(v)$. Then the set $\textrm{ShortPath}_{X_f}(v) -Y_k$ must contain at least three different connected components, each consisting of points with a value of *f* less than $\pi (v)$. Hence, for any $\epsilon$ sufficiently small, we have that the map $\pi _0(f^{-1}(-\infty ,t-\epsilon ]) \rightarrow \pi _0(f^{-1}(-\infty ,t])$, is at least three-to-one at some point in its image. Since path connectedness and connectedness are identical notions for subsets of geometric trees, this contradicts that *T* is binary. So $Y_k$ must be connected. $\square$

## Retraction of the fiber to configuration space on a tree

Let $X$ be a geometric tree. We metrise spaces of functions on *X* via the supremum norm. In this section we fix a generic labelled cellular merge tree $T$ and we analyse the subspace of functions *f* in the fiber:

$$\begin{aligned} \textrm{MT}^{-1}(T)= \bigg \{f:X\rightarrow \mathbb {R}, \, \textrm{MT}(f) \text { is isomorphic to } T\bigg \}. \end{aligned}$$

It follows from Theorem 9 that every $f\in \textrm{MT}^{-1}(T)$ has finitely many local minima. As in the previous section, we take a cellular structure for $T$ that has only branch points and leaves as nodes. We will simplify $\textrm{MT}^{-1}(T)$ by means of a series of homotopy equivalences

$$\begin{aligned} \textrm{MT}^{-1}(T){\mathop {{\longrightarrow }}\limits ^{\sim }}\textrm{Conf}_{\textrm{Crit}}(X,T) {\mathop {{\longrightarrow }}\limits ^{\sim }}\textrm{Conf}_{\textrm{Min}}(X,T) {\mathop {{\longrightarrow }}\limits ^{\sim }}\textrm{Conf}(X,T), \end{aligned}$$

where the spaces $\textrm{Conf}_{\textrm{Crit}}(X,T)$, $\textrm{Conf}_{\textrm{Min}}(X,T)$, and $\textrm{Conf}(X,T)$ are configuration spaces tracking the local minima and saddles of a function $f\in \textrm{MT}^{-1}(T)$. We will define the spaces $\textrm{Conf}_{\textrm{Crit}}(X,T)$, $\textrm{Conf}_{\textrm{Min}}(X,T)$, and $\textrm{Conf}(X,T)$ shortly, but first we must make a couple convenient definitions.

Consider any subset $\tilde{X} = (X_1,\ldots ,X_n) \subseteq X^n$. Motivated by the definition we made in Proposition 18, for a node $v$ of $T$, possibly a leaf, we define:

$$\begin{aligned}\textrm{Conv}_{\tilde{X}}(v):=\textrm{Conv}\Big \{X_i \mid \text { leaf } l_i \text { is a descendant of }v\Big \}\subseteq X.\end{aligned}$$

In particular, given a function *f* with finitely many local minima, and $X_f = (X_1,\ldots ,X_n)$ its collection of local minima, with $X_i$ corresponding to leaf $l_i$ as in Proposition 18, this definition agrees with our previous definition of $\textrm{Conv}_{X_f}(v)$. Also note that for any leaf $l_i$, we have $\textrm{Conv}_{\tilde{X}}(l_i) = X_i$. We define $\textrm{Conv}_x(v)$ for $x = (x_1,\ldots ,x_n)\in X^n$ similarly as

$$\begin{aligned} \textrm{Conv}_{x}(v):=\textrm{Conv}\Big \{x_i \mid \text { leaf } l_i \text { is a descendant of }v\Big \}\subseteq X. \end{aligned}$$

**Fig. 3:**
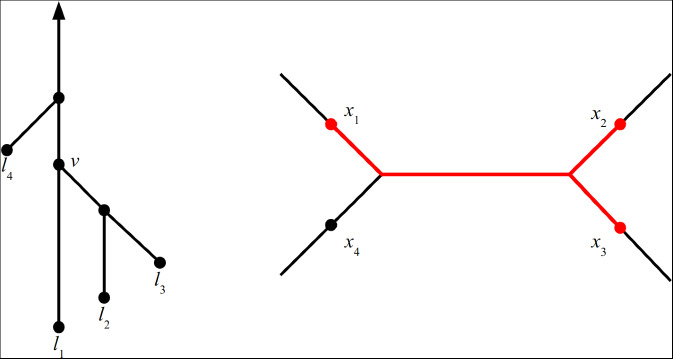
(Left) A cellular merge tree $T$ with four leaves. (Right) An element $x = (x_1,x_2,x_3,x_4)$ of $\textrm{Conf}(X,T) \subseteq \textrm{Conf}_4(X)$, for $X$ a geometric tree. Here, the set $\textrm{Conv}_x(v)$ associated to the node $v\in T$ is highlighted. It consists of the union of the shortest path from $x_1$ to $x_2$ and the shortest path from $x_2$ to $x_3$.

We illustrate this definition in Figure 3. These definitions will be a useful shorthand for characterising the spaces $\textrm{Conf}_{\textrm{Crit}}(X,T), \textrm{Conf}_{\textrm{Min}}(X,T)$, and $\textrm{Conf}(X,T)$. The last of these spaces is the most important for this paper.

### Definition 19

Denote the usual ordered configuration space on *n* points by $\textrm{Conf}_n(X)$. This is the topological space

$$\begin{aligned} \textrm{Conf}_n(X) := \{x = (x_1,\ldots , x_n) \in X^n: x_i \ne x_j \text { for all }i\ne j\}. \end{aligned}$$

It is topologised via the subspace topology of $X^n$. The space $\textrm{Conf}(X,T)$ is given by

4 $$\begin{aligned} \begin{aligned} \textrm{Conf}(X,T):=\bigg \{&x = (x_1,\cdots ,x_n) \in \textrm{Conf}_n(X) \mid \\&\forall \text { nodes } v,v'\in T, \;\textrm{Conv}_x( v) \cap \textrm{Conv}_x( v') \ne \emptyset \Rightarrow v\preceq v' \text { or } v'\preceq v\bigg \}. \end{aligned} \end{aligned}$$

A configuration $(x_1,\cdots ,x_n)\in \textrm{Conf}(X,T)$ consists of points $x_i\in X$ such that there exists a function *f* with merge tree $T$ that has local minimum $\{x_i\}$ corresponding to the leaf $l_i$ of *T* for each *i*. While useful for intuition, this fact is not obvious, and follows from the proof of the upcoming Theorem 20. Moreover this fact is not needed at a technical level in our paper. The reason we choose the above definition for the space $\textrm{Conf}(X,T)$ is that the characterisation in terms of intersections of sets $\textrm{Conv}_x(v)$ will be useful for finding paths in $\textrm{Conf}(X,T)$ in the following section. Because in general a function *f* with merge tree $T$ could achieve its minima on arbitrary closed sets rather than points, it is natural to extend $\textrm{Conf}(X,T)$ to the following configuration space of closed sets:

5 $$\begin{aligned} \begin{aligned} \textrm{Conf}_{\textrm{Min}}(X,T) :=&\bigg \{\tilde{X}=(X_1,\cdots ,X_n) \subseteq X^n \mid X_i\text { disjoint connected closed sets,}\\&\forall \text { nodes } v,v'\in T, \;\textrm{Conv}_{\tilde{X}}( v) \cap \textrm{Conv}_{\tilde{X}}( v') \ne \emptyset \Rightarrow v\preceq v' \text { or } v'\preceq v\bigg \}. \end{aligned} \end{aligned}$$

Note that a set configuration $\tilde{X}=(X_1,\cdots ,X_n)\in \textrm{Conf}_{\textrm{Min}}(X,T)$ gives rise to convex hulls $\textrm{Conv}_{\tilde{X}}(v)$ for any node $v\in T$. For $v$ an internal node with children $Lv$ and $Rv$, we will also consider the following subset of *X*:

$$\begin{aligned}\textrm{ShortPath}_{\tilde{X}}(v) := \textrm{ShortPath}\big (\textrm{Conv}_{\tilde{X}}(Lv),\textrm{Conv}_{\tilde{X}}(Rv)\big ). \end{aligned}$$

We are now ready to introduce our last configuration space $\textrm{Conf}_{\textrm{Crit}}(X,T)$ of closed sets where we also record saddles of a function $g\in \textrm{MT}^{-1}(T)$:

6 $$\begin{aligned} \begin{aligned} \textrm{Conf}_{\textrm{Crit}}(X,T) :=&\bigg \{(\tilde{X},\tilde{Y})=(X_1,\cdots ,X_n,Y_1,\cdots , Y_{n-1}) \subseteq X^{2n-1}\mid \\&X_i, Y_j\text { disjoint connected closed sets}, \\&(X_1,\cdots ,X_n)\in \textrm{Conf}_{\textrm{Min}}(X,T),\\&\forall j, \, Y_j \cong [0,1], \, Y_j \text { is a subset of the interior of } \textrm{ShortPath}_{\tilde{X}}(v_j) \bigg \}. \end{aligned} \end{aligned}$$

Note that the Hausdorff distance inherited from any metric on *X* compatible with its CW structure induces topologies on configuration spaces of closed sets, hence subset topologies for our spaces $\textrm{Conf}_{\textrm{Crit}}(X,T),\textrm{Conf}_{\textrm{Min}}(X,T)$ and $\textrm{Conf}(X,T)$.

### Theorem 20

The spaces $\textrm{MT}^{-1}(T), \textrm{Conf}_{\textrm{Crit}}(X,T), \textrm{Conf}_{\textrm{Min}}(X,T)$, and $\textrm{Conf}(X,T)$ are homotopy equivalent.

The proof of Theorem 20 decomposes as the construction of three consecutive homotopy equivalences:

$$\begin{aligned} \textrm{MT}^{-1}(T){\mathop {{\longrightarrow }}\limits ^{\sim }}\textrm{Conf}_{\textrm{Crit}}(X,T) {\mathop {{\longrightarrow }}\limits ^{\sim }}\textrm{Conf}_{\textrm{Min}}(X,T) {\mathop {{\longrightarrow }}\limits ^{\sim }}\textrm{Conf}(X,T). \end{aligned}$$

### Proof

For the proof we will fix a metric *d* on *X*.

### Step 1: ${\textrm{Conf}_{\textrm{Min}}(X,T){\mathop {{\longrightarrow }}\limits ^{\sim }}\textrm{Conf}(X,T)}$

We choose an arbitrary leaf $\tau \in X$, i.e. the image of a leaf vertex in a cellular decomposition of *X*. For any connected closed subset $A\subseteq X$, the point $\tau (A)$ which is closest to $\tau$ is uniquely defined. We can continuously contract *A* to $\tau (A)$ with a family $(A_t)_{0\le t \le 1}$ of enclosed subsets:

$$\begin{aligned}A_t:= \big \{ a\in A \mid d(a,\tau (A))\le (1-t)\textrm{diam}(A)\big \}.\end{aligned}$$

Note that the existence of a contraction of this sort is dependent on the tree structure of *X*. In particular, for other choices of *X* (e.g. geometric realizations of graphs that are not trees) it may be the case that *a* is not uniquely defined and moreover the contraction written above may not consist of connected sets.

Given a configuration of connected closed sets $(X_1,\cdots , X_n)$, the continuous contraction of each $X_i$ to $\tau (X_i)$ defines a deformation retract of $\textrm{Conf}_{\textrm{Min}}(X,T)$ to $\textrm{Conf}(X,T)$:

$$\begin{aligned}\textrm{H}: \big (t,(X_1,\cdots ,X_n)\big )\in [0,1]\times \textrm{Conf}_{\textrm{Min}}(X,T)\longmapsto ((X_1)_t,\cdots ,(X_n)_t)\in \textrm{Conf}_{\textrm{Min}}(X,T)\end{aligned}$$

Indeed, under this map, any $\tilde{X}\in \textrm{Conf}_{\textrm{Min}}(X,T)$ is mapped to an element $\textrm{H}(t,\tilde{X})$ that satisfies the condition for being in $\textrm{Conf}_{\textrm{Min}}(X,T)$: for any nodes $v,v'\in T$, if $\textrm{Conv}_{\textrm{H}(t,\tilde{X})}(v)\cap \textrm{Conv}_{\textrm{H}(t,\tilde{X})}(v')\ne \emptyset$, then we also have $\textrm{Conv}_{\tilde{X}}(v)\cap \textrm{Conv}_{\tilde{X}}(v')\ne \emptyset$, and so $v\preceq v'$ or $v'\preceq v$, as desired, since $\textrm{Conv}_{\textrm{H}(t,\tilde{X})}(v)\subseteq \textrm{Conv}_{\tilde{X}}(v)$ and $\textrm{Conv}_{\textrm{H}(t,\tilde{X})}(v')\subseteq \textrm{Conv}_{\tilde{X}}(v')$.

#### Step 2: $\textrm{Conf}_{\textrm{Crit}}(X,T) {\mathop {{\longrightarrow }}\limits ^{\sim }}\textrm{Conf}_{\textrm{Min}}(X,T)$

Let $\tilde{X}=(X_1,\cdots ,X_n)\in \textrm{Conf}_{\textrm{Min}}(X,T)$ be a configuration of closed sets. Given $1\le j \le (n-1)$, we have that $\textrm{ShortPath}_{\tilde{X}}(v_j)$ is a closed segment $[a_j,b_j]$ in *X*, where by convention the extreme $a_j$ lies in $\textrm{Conv}_{\tilde{X}}(Lv)$.

Let (*s*, *t*) be an element of the open standard simplex $\Delta _2:=\{(s,t)\mid 0<s\le t<1\}$. Define

$$\begin{aligned}Y_j^{s,t}:=\bigg \{ x\in [a_j,b_j] \mid s \le \frac{d(a_j,x)}{d(a_j,b_j)} \le t \bigg \}.\end{aligned}$$

Note that, for varying $1\le j \le (n-1)$, the interiors of the shortest paths $\textrm{ShortPath}_{\tilde{X}}(v_j)$ (meaning these shortest paths without their two endpoints) are disjoint from each other. Indeed, let’s assume, seeking contradiction, that the interior of $\textrm{ShortPath}_{\tilde{X}}(v)$ intersects the interior of $\textrm{ShortPath}_{\tilde{X}}(v')$ for some distinct nodes $v,v'\in T$. We then have $\textrm{Conv}_{\tilde{X}}(v)\cap \textrm{Conv}_{\tilde{X}}(v')\ne \emptyset$, and since $\tilde{X}\in \textrm{Conf}_{\textrm{Min}}(X,T)$, we can assume without loss of generality that $v$ is a descendant of $v'$. But then $\textrm{Conv}_{\tilde{X}}(v)$ is disjoint from the interior of $\textrm{ShortPath}_{\tilde{X}}(v')$, and since $\textrm{ShortPath}_{\tilde{X}}(v)\subseteq \textrm{Conv}_{\tilde{X}}(v)$, we reach the contradiction that the interiors of the shortest paths $\textrm{ShortPath}_{\tilde{X}}(v)$ and $\textrm{ShortPath}_{\tilde{X}}(v')$ do not intersect. Therefore the sets $Y_j^{s_j,t_j}$ are disjoint from each other and from the sets $X_i$, and we have the homeomorphism:

$$\begin{aligned} [(X_i)_{i=1}^n, (Y_j^{s_j,t_j})_{j=1}^{n-1}] \in&\textrm{Conf}_{\textrm{Crit}}(X,T)\\&\longmapsto [(X_i)_{i=1}^n, (s_j,t_j)_{j=1}^{n-1}]\in \textrm{Conf}_{\textrm{Min}}(X,T) \times (\Delta _2)^{n-1}. \end{aligned}$$

The deformation retract of each factor of $\Delta _2$ to a point gives us the homotopy equivalence from $\textrm{Conf}_{\textrm{Crit}}(X,T)$ to $\textrm{Conf}_{\textrm{Min}}(X,T)$.

#### Step 3: $\textrm{MT}^{-1}(T){\mathop {{\longrightarrow }}\limits ^{\sim }}\textrm{Conf}_{\textrm{Crit}}(X,T)$

**Fig. 4:**
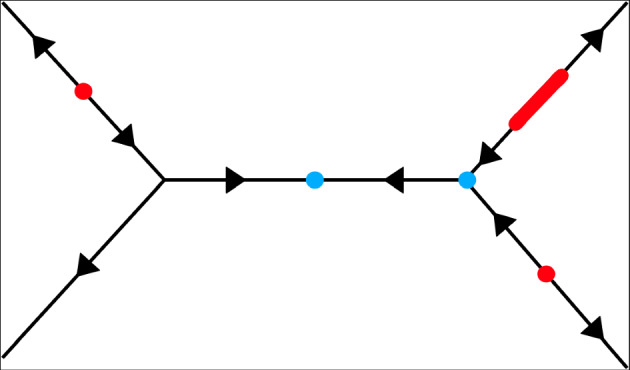
The local minima and saddles of a function on a tree. Directions of arrows on the depicted tree *X* indicate where the function is increasing. The three red regions indicate the locations of local minima $X_i(f)$ while the two blue regions indicate the locations of saddles $Y_j(f)$. Saddles do not need to be local maxima: in this example, one saddle is a local maximum while the other is not (colour figure online)

To show that there is a homotopy equivalence between these two spaces, we will need to define a map which sends a function *f* to its local minima and saddles, see Figure 4.

For $f\in \textrm{MT}^{-1}(T)$ and $1\le i \le n$, let $X_i(f)$ denote the connected subset of *X* where *f* achieves the minimum $m_i$ corresponding to leaf $l_i$ of $T$. In this proof we write $X_i(f)$ to indicate that this set changes as *f* varies. The map $f\mapsto X_i(f)$ is continuous.

Let $X_f = (X_1(f), \ldots , X_n(f))$ and let $v,v'\in T$ be two nodes such that $A:=\textrm{Conv}_{X_f}(v)\cap \textrm{Conv}_{X_f}(v')\ne \emptyset$. We assume without loss of generality that $\pi (v)\le \pi (v')$ and show that $v$ is a descendant of $v'$, i.e. $v\preceq v'$.

Since $f\in \textrm{MT}^{-1}(T)$, there is an isomorphism $\phi :T \rightarrow \textrm{MT}(f)$. Solely for the sake of proving that $v\preceq v'$, we let $C(v) \subseteq X$ denote the set of

$$\begin{aligned} C(v) := \{x\in X: (x,\pi (v)) \text { is a representative of } \phi (v) \in \textrm{MT}(f) \} \end{aligned}$$

It follows from this definition that $C(v)$ is a connected component of $f^{-1}(-\infty ,\pi (v)]$. Moreover, for nodes *w* and $w'$ of *T* we have $C(w) \subseteq C(w')$ if and only if $w\preceq w'$.

For any leaf $l_i\preceq v$, Lemma 12 implies that $C(l_i)$ is a local minimum. Since $f ( C(l_i)) = \pi (l_i)$, and $C(l_i)$ is a local minimum, $C(l_i) = X_i(f)$. Therefore $X_i(f) = C(l_i) \subseteq C(v)$. Hence, whenever $l_i,l_j\preceq v$, the set $\textrm{ShortPath}(X_i(f),X_j(f))$ is contained in *C*(*v*) since *C*(*v*) is a connected subset of a geometric tree and hence path connected. It follows that $C(v)$ must contain $\textrm{Conv}_{X_f}(v)$. Similarly, $C(v')$ must contain $\textrm{Conv}_{X_f}(v')$. So both $C(v)$ and $C(v')$ contain *A*. Since *C*(*v*) is a connected component of $f^{-1}(-\infty ,\pi (v)]$, $C(v')$ is a connected component of $f^{-1}(-\infty ,\pi (v')]$, and $\pi (v)\le \pi (v')$, this implies $C(v) \subseteq C(v')$. So $v \preceq v'$. Therefore $X_f \in \textrm{Conf}_{\textrm{Min}}(X,T)$.

By Proposition 18, for $1\le j \le n-1$, the restriction of *f* to $\textrm{ShortPath}_{\tilde{X}}(v_j)$ attains its maximum $\pi (v_j)$ on a unique connected closed set $Y_j(f)$. Since local minima $X_i(f)$ vary continuously with *f*, so do the convex hulls $\textrm{Conv}_{\tilde{X}}(v_j)$ and the shortest paths $\textrm{ShortPath}_{\tilde{X}}(v_j)$ between them, and therefore the maps $f\mapsto Y_j(f)$ are continuous, and so we have defined a continuous map:

$$\begin{aligned} F: \textrm{MT}^{-1}(T)&\longrightarrow \textrm{Conf}_{\textrm{Crit}}(X,T)\\ f&\longmapsto \big (X_1(f),\cdots ,X_n(f),Y_1(f),\cdots ,Y_{n-1}(f)\big ). \end{aligned}$$

To show that *F* is a homotopy equivalence, we define a map in the other direction. Let $Z=(\tilde{X},\tilde{Y})=(X_1,\cdots ,X_n,Y_1,\cdots ,Y_{n-1})\in \textrm{Conf}_{\textrm{Crit}}(X,T)$. We construct a function $f_Z$ by induction on the height of the nodes of $T$. To begin with, we define $f_Z(X_i):= m_i$. Next, let $v_j\in T$ be a node such that $f_Z$ is already defined on $\textrm{Conv}_{\tilde{X}}(Lv_j)$ and $\textrm{Conv}_{\tilde{X}}(Rv_j)$. We extend $f_Z$ to

$$\begin{aligned}\textrm{Conv}_{\tilde{X}}(v_j)=\textrm{Conv}_{\tilde{X}}(Lv_j)\cup \textrm{Conv}_{\tilde{X}}(Rv_j)\cup \textrm{ShortPath}_{\tilde{X}}(v_j),\end{aligned}$$

by letting $f_Z(Y_j):= \pi (v_k)$ and by linear interpolation on the rest of $\textrm{ShortPath}_{\tilde{X}}(v_j)$. Explicitly, let *L* be the length of $\textrm{ShortPath}_{\tilde{X}}(v_j)$. There is a unique isometry

$$\begin{aligned}h : [0,L] \rightarrow \textrm{ShortPath}_{\tilde{X}}(v_j)\end{aligned}$$

such that $h(0) \in \textrm{Conv}_{\tilde{X}}(Lv_j)$ and $h(L) \in \textrm{Conv}_{\tilde{X}}(Rv_j)$. We define a function $g_j:[0,L] \rightarrow \mathbb {R}$ by $g_j(0) = f \circ h^{-1}(0)$, $g_j(L) = f \circ h^{-1}(0)$, and $g_j(h^{-1}(Y_j)) = \pi (v_j)$. This defines $g_j$ on three closed connected disjoint subsets of [0, *L*]. On the remainder of [0, *L*] we define $g_j$ by linear interpolation. Thus we can extend $f_Z$ to be defined on $\textrm{ShortPath}_{\tilde{X}}(v_j)$ via $f_Z:= g\circ h^{-1}$.

At the end of this process, repeated at each branch point of *T*, $f_Z$ is defined on the convex hull $K:= \textrm{Conv}\{X_1(f),\ldots ,X_n(f)\}$, outside of which we let $f_Z$ increase in all directions:

$$\begin{aligned}&\forall x\in X\setminus K,\\ \qquad f_Z(x):= f_Z(&\textrm{proj}_{K}(x))+ d(x,K). \end{aligned}$$

Here, $\textrm{proj}_{K}(x)$ is equal to *x* if $x\in K$ and otherwise is the closest point to *x* in *K*. By Proposition 18 $\textrm{MT}(f_Z)$ is isomorphic to *T* as desired.

This gives us a continuous map:

$$\begin{aligned}G: Z\in \textrm{Conf}_{\textrm{Crit}}(X,T) \longmapsto f_Z\in \textrm{MT}^{-1}(T),\end{aligned}$$

and clearly $F\circ G= \textrm{Id}$. Conversely, by Proposition 18, the straight-line interpolation

$$\begin{aligned}(t,f)\in [0,1]\times \textrm{MT}^{-1}(T)\longmapsto (1-t)f +t G\circ F(f)\end{aligned}$$

is valued in $\textrm{MT}^{-1}(T)$, hence it defines a homotopy equivalence $G\circ F\sim \textrm{Id}$. $\square$

## The topology of functions on a tree with a given barcode

Let $X$ be a geometric tree. Since subsets of geometric trees are component-wise contractible, the barcodes $\textrm{PH}_i(f)$ for $i\ge 1$ are all empty for any $f:X\rightarrow \mathbb {R}$. So in the rest of the section we identify the set of barcodes $\textrm{PH}(f)=\{\textrm{PH}_i(f)\}_{i\ge 0}$ with $\textrm{PH}_0(f)$, the barcode of the zero dimensional persistent homology of *f*. Similarly, instead of using the notation *D* for the set of all barcodes $\{D_i\}_{i\ge 0}$, we identify *D* with $D_0$, the single non-empty barcode. Hence we will refer to $\textrm{PH}_0^{-1}(D_0)$ simply as $\textrm{PH}^{-1}(D)$.

In this section we fix a barcode *D* and analyse the space $\textrm{PH}^{-1}(D)$ of continuous pfd functions with barcode *D*. In the rest of the section we assume that *D* is generic in the following sense:

### Definition 21

A barcode *D* is *generic* if it is finite and all its interval endpoints are distinct.

As observed in Section 2.4, computing the fiber $\textrm{PH}^{-1}(D)$ can be solved by computing the fibers of two composite maps:Since *D* is finite, we know that $\textrm{MT}(f)$ is cellular if $\textrm{PH}(f) = D$ by Lemma 7 and Theorem 9 combined. This justifies our use of the notation *T* above. This fact also means that the fiber of the second map is known from (Curry 2018): the number of cellular merge trees (up to isomorphism) giving rise to a generic barcode *D* is finite and computed in (Curry 2018, Theorem 4.8). Therefore it remains to analyse functions with a given cellular merge tree $T$ (up to isomorphism).

We first identify $\textrm{MT}^{-1}(T)$ with a connected component of $\textrm{PH}^{-1}(D)$ in subsection 6.1, and then, in subsection 6.2, we derive some topological properties of these components. Before we begin we first record the following two useful properties.

### Proposition 22

If *D* is a generic barcode and $f\in \textrm{PH}^{-1}(D)$, then the merge tree $\textrm{MT}(f)$ is a generic cellular merge tree.

### Proof

Since *D* is finite, by Lemma 7, *f* has finitely many local minima, and in turn $(\textrm{MT}(f),\pi _f)$ is cellular by the first assertion of Theorem 9. Since $\textrm{PH}(\pi _f)=\textrm{PH}(f)$ has distinct interval endpoints, no more than two connected subsets can merge at a time in the sublevel-sets of $\pi _f$, hence $\textrm{MT}(f)$ is a binary tree. Similarly, no two leaves of $\textrm{MT}(f)$ can have the same value of $\pi _f$, as this would force $\textrm{PH}(f)$ to have a repeated left endpoint. $\square$

### Proposition 23

Given a geometric tree $X\ne \emptyset$ and a cellular merge tree $T$, the fiber $\textrm{MT}^{-1}(T)$ and the configuration space $\textrm{Conf}(X,T)$ are non-empty.

### Proof

A function on the unit interval (hence more generally on any non-empty tree $X$) with merge tree $T$ can be constructed, e.g. as in (Curry 2018, Proposition 6.8). Therefore $\textrm{MT}^{-1}(T)\ne \emptyset$, and by Theorem 20, $\textrm{Conf}(X,T)\ne \emptyset$ as well. $\square$

### Counting connected components in the fiber over binary trees

Recall that in this section we assume the barcode *D* is generic. Recall that we have identified *D* with $D_0$, thereby avoiding trivial cases where *D* has intervals in degree greater than 0, in which case we have $\textrm{PH}^{-1}(D)= \emptyset$. We further assume that *D* has exactly one unbounded interval $[b,\infty )$ that contains all other intervals, as when this does not hold we again have $\textrm{PH}^{-1}(D)= \emptyset$.

#### Proposition 24

Given $T$ a generic cellular merge tree with barcode *D*, $\textrm{MT}^{-1}(T)$ is a non-empty union of connected components in $\textrm{PH}^{-1}(D)$.

#### Proof

 $\textrm{MT}^{-1}(T)\ne \emptyset$ (Proposition 23) and $\textrm{MT}$ is locally constant (up to isomorphism) on $\textrm{PH}^{-1}(D)$ (Proposition 15). $\square$

#### Theorem 25

Let *X* be a geometric tree not homeomorphic to the unit interval. Given a generic cellular merge tree $T$, the fiber $\textrm{MT}^{-1}(T)$ is nonempty and path-connected. In particular, if $f\in \textrm{MT}^{-1}(T)$ has barcode *D*, then $\textrm{MT}^{-1}(T)$ is the path connected component of the fiber $\textrm{PH}^{-1}(D)$ containing *f*.

The homotopy type of $\textrm{PH}^{-1}(D)$ is already known for the case where *X* is homeomorphic to the unit interval. If *D* has *n* intervals, the number of path components in $\textrm{PH}^{-1}(D)$ is known to be

$$\begin{aligned} \# \pi _0(\textrm{PH}^{-1}(D))=2^{n-1}\prod _{\begin{array}{c} [b,d)\in D\\ d \ne \infty \end{array}} \# \big \{ [b',d') \in D \mid [b,d) \subset [b',d')\big \}. \end{aligned}$$

from (Curry 2018) and these path components are known to be contractible from (Leygonie and Beers 2022).

The proof mainly relies on the following result (recall Definition 19) .

#### Theorem 26

Let *X* be a geometric tree not homeomorphic to the unit interval. Let $T$ be a generic cellular merge tree. Then $\textrm{Conf}(X,T)$ is path-connected.

Before proving the Theorem 26, let us see how it leads to Theorem 25.

#### Proof of Theorem 25

From Proposition 23, $\textrm{MT}^{-1}(T)\ne \emptyset$, and from Theorem 20, $\textrm{MT}^{-1}(T)$ is homotopy equivalent to $\textrm{Conf}(X,T)$, which is path-connected by Theorem 26. If $T$ has barcode *D*, by Proposition 24, $\textrm{MT}^{-1}(T)$ is a non-empty union of connected components of $\textrm{PH}^{-1}(D)$. Therefore it equals exactly one such connected component. $\square$

To prove that $\textrm{Conf}(X,T)$ is path-connected, we proceed in two steps, each relying on a key lemma. First, we show how to deform a configuration of points into one whose points all lie on a common edge of *X*. The main result to achieve this step is Lemma 29. Then, given two configurations whose points lie on an edge, we show how to connect them using a branch point of *X*. We achieve this step with Lemma 34. Throughout, we fix a labelling of the cellular merge tree $T$.

The following two results, Lemma 27 and Lemma 28, will be used repeatedly during our argument.

#### Lemma 27

Let $x = (x_1, \ldots ,x_n)$ be in $\textrm{Conf}(X,T)$, and $x' = (x_1,\ldots ,x_{i-1},y,x_{i+1},\ldots ,x_n)$ be in $\textrm{Conf}_n(X)$. Fix $P\subseteq X$, the image of some path from $x_i$ to *y*. If $\textrm{Conv}_x(v)$ does not intersect *P* for any $v$ that is not an ancestor of $l_i$, then $x'\in \textrm{Conf}(X,T)$ and there is a path from *x* to $x'$ in $\textrm{Conf}(X,T)$.

#### Proof

For $p\in P$, let $x(p) = (x_1,\ldots , x_{i-1}, p, x_{i+1},\ldots ,x_n)$. Let $v,v'$ be nodes of *T* with neither $v\preceq v'$ nor $v'\preceq v$. It cannot be the case that both $v$ and $v'$ are ancestors of $l_i$ as ancestors of $l_i$ are totally ordered. If neither $v$ nor $v'$ are ancestors of $l_i$ then

$$\begin{aligned} \textrm{Conv}_{x(p)}(v)\cap \textrm{Conv}_{x(p)}(v') = \textrm{Conv}_x(v)\cap \textrm{Conv}_x(v') = \emptyset , \end{aligned}$$

with the final equality following from the fact that $x\in \textrm{Conf}(X,T)$. Lastly, consider the case where either $v$ or $v'$ is an ancestor of $l_i$, but not both. Without loss of generality assume $v'$ is an ancestor of $l_i$ and $v$ is not. Note that

$$\begin{aligned} \textrm{Conv}_{x(p)}(v')\subseteq \textrm{Conv}_x(v')\cup \textrm{ShortPath}(x_i,p) \subseteq \textrm{Conv}_x(v')\cup P. \end{aligned}$$

Thus, since $\textrm{Conv}_x(v)\cap \textrm{Conv}_x(v') = \emptyset$, we have

$$\begin{aligned} \textrm{Conv}_{x(p)}(v)\cap \textrm{Conv}_{x(p)}(v') \subseteq \textrm{Conv}_x(v)\cap \big (\textrm{Conv}_x(v')\cup P\big ) = \textrm{Conv}_x(v)\cap P = \emptyset . \end{aligned}$$

Thus the criteria for *x*(*p*) to be an element of $\textrm{Conf}(X,T)$ are satisfied for all $p\in P$. The result follows. $\square$

#### Lemma 28

Let $x = (x_1,\ldots , x_n) \in \textrm{Conf}(X,T)$. If $x_i$ is a branch point with incident edge *e* for some *i*, then sufficiently short paths from $x_i$ to points *y* in the interior of *e* define paths from *x* to $(x_1,\ldots , x_{i-1},y,x_{i+1}, \ldots , x_n)$ in $\textrm{Conf}(X,T)$.

#### Proof

Let *y* be sufficiently close to $x_i$ that there is no $x_j$, where $j\ne i$, and no additional branch point in the shortest path from *y* to $x_i$. Denote this path by *P* and let $x' = (x_1,\ldots , x_{i-1},y,x_{i+1}, \ldots , x_n)$. For $v\in T$ a node not ancestral to $l_i$, the set $\textrm{Conv}_x(v)$ does not intersect *P*, as doing so would mean it would contain $x_i$. The result follows by Lemma 27. $\square$

#### Lemma 29

Fix any $x = (x_1,\ldots ,x_n)\in \textrm{Conf}(X,T)$. There is an edge *e* in *X* and a path from *x* to $x' = (x'_1,\ldots ,x'_n)$ in $\textrm{Conf}(X,T)$ where every $x'_i$ is in the interior of *e*.

#### Proof

**Fig. 5:**
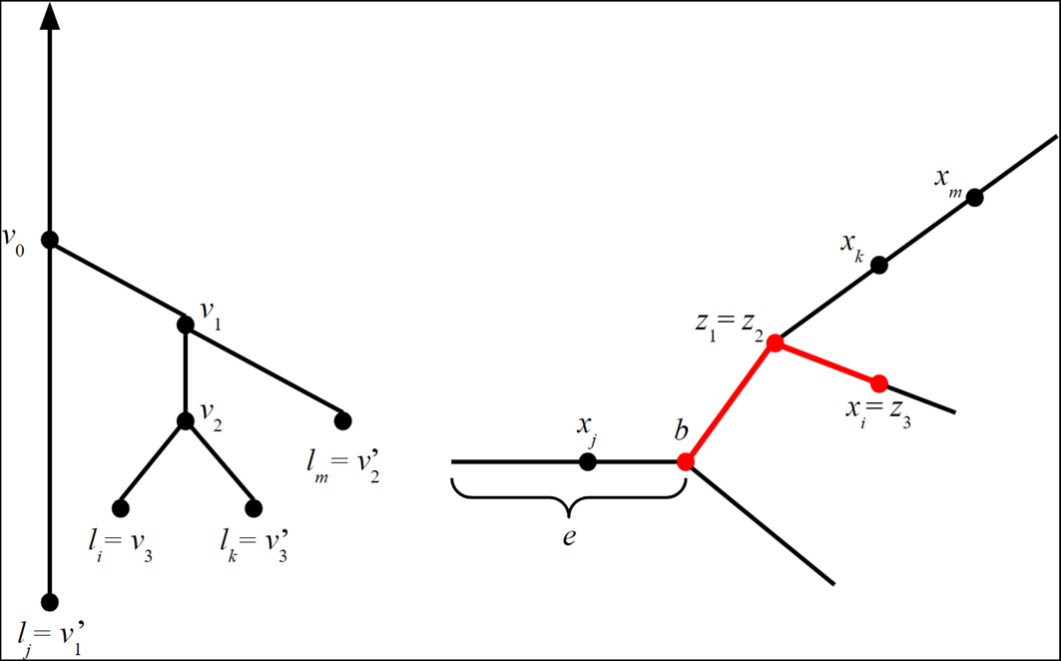
A merge tree $T$ (left) and a sample geometric tree *X* (right) illustrating the main construction of Lemma 29. Highlighted in red is the path constructed from from a point $x_i$ in *X* to *b*, which lifts to a path in $\textrm{Conf}(X,T)$. Note that in this example we could have also constructed a path from *b* to $x_{k}$, but not to $x_{m}$, because $\textrm{Conv}_x(v_2)$ interrupts the path from *b* to $\textrm{Conv}_x(v_2')$ (colour figure online)

Using Lemma 28, we assume without loss of generality that no point of *x* is on a branch point or leaf of *X*. Hence each point of *x* lies in the interior of an edge of *X*. If every point of *x* lies in the interior of the same edge *e*, then we are done. Therefore, assume otherwise and let $e\in X$ be an edge with some but not all entries of *x* in its interior. We will build a path to an $x'$ with one more entry in *e*, giving us the lemma by induction. The construction of the path is illustrated in Figure 5.

By hypothesis, there is an entry of *x* in at least one of the two path components of *X* minus the interior of *e*. Let *b* denote the endpoint of *e* in this path component, which we will call *C*. Let $x_j$ denote the entry of *x* in the interior of *e* that is closest to *b*. We will construct a path from *b* to some $x_i\in C$ and show that this path lifts to a path in $\textrm{Conf}(X,T)$, using Lemma 27.

Let $v_0\in T$ be the least ancestor of $l_j$ such that $\textrm{Conv}_x(v_0)$ contains *b*. Thus $v_0$ is not $l_j$ itself, so $v_0$ has two children, $v_1$ and $v'_1$, exactly one of which is an ancestor of $l_j$. Without loss of generality, suppose $v'_1$ is an ancestor of $l_j$ and $v_1$ is not. Let *P* denote the shortest path in *X* from *b* to $\textrm{Conv}_x(v_1)$, and let $z_1$ denote the endpoint of *P* intersecting $\textrm{Conv}_x(v_1)$. Since both *b* and $z_1$ are in $\textrm{Conv}_x(v_0)$, so is *P*. If *b* is in $\textrm{Conv}_x(v_1)$, then *P* consists of the singleton *b* and does not intersect $\textrm{Conv}_x(v'_1)$. Otherwise, *b* is in neither $\textrm{Conv}_x(v_1)$ nor $\textrm{Conv}_x(v'_1)$. Thus *b* lies on the shortest path between these two sets. Hence *P* is a subset of this shortest path, and therefore does not intersect $\textrm{Conv}_x(v'_1)$. So *P* does not intersect $\textrm{Conv}_x(v'_1)$ in either case.

We will continue to augment *P* until it has reached some $x_i$. Inductively, assume we have already constructed a path *P* from *b* to a point $z_{m-1}$ in $\textrm{Conv}_x(v_{m-1})$. If $v_{m-1}$ has no children, then $\textrm{Conv}_{x}(v_{m-1})$ is a singleton containing $x_i$ for some *i*, and we are done. Otherwise, let $v_m$ and $v'_m$ denote the children of $v_{m-1}$.

Either the shortest path from $z_{m-1}$ to $\textrm{Conv}_x(v_m)$ does not intersect $\textrm{Conv}_x(v'_m)$ or the shortest path from $z_{m-1}$ to $\textrm{Conv}_x(v'_m)$ does not intersect $\textrm{Conv}_x(v_m)$. Without loss of generality, assume we are in the first case. Since $z_{m-1}$ is in $\textrm{Conv}_x(v_{m-1})$, so is the shortest path from $z_{m-1}$ to $\textrm{Conv}_x(v_m)$. We augment *P* by this path and refer to its augmented endpoint as $z_m$. Since $v_0$ has finitely many descendants, this process is guaranteed to terminate eventually, and we will obtain a path *P* from *b* to some $x_i$. Further, since every augmented portion of *P* lies in $\textrm{Conv}_x(v_m)$ for some *m*, all of *P* lies in $\textrm{Conv}_x(v_0)$.

Let $v$ be a node of $T$ not ancestral to $l_i$. If $\textrm{Conv}_x(v)$ intersects *P* then it intersects $\textrm{Conv}_x(v_0)$, which contains *P*. Therefore either $v\preceq v_0$ or $v_0\preceq v$. It cannot be that $v_0\preceq v$ since $l_i$ is not a descendant of $v$, so $v\preceq v_0$. Again since $l_i$ is not a descendant of $v$, $v$ cannot be $v_m$ for any *m*, so $v$ is a descendant of some $v'_m$. However, by construction, *P* does not intersect $\textrm{Conv}_x(v'_m)$ for any *m*, so it cannot intersect $\textrm{Conv}_x(v)$ either. Applying Lemma 27 allows us to move $x_i$ onto *b*. Then applying Lemma 28, we can further move $x_i$ into the interior of *e*. By induction it follows that we can move every point $x_1,\ldots ,x_n$ into the interior of *e*. $\square$

Now that we know we can get all points onto one edge, we want to be able to move groups of points along curves. Intuitively, once every point is on some curve, we should be able to slide the points along this curve freely, provided we never change their order, as this would require points to cross. Let $x = (x_1,\ldots ,x_n)\in \textrm{Conf}_n(X)$, here $\textrm{Conf}_n(X)$ is the usual ordered configuration space of *n* points on *X*, and suppose that there is a subset $Y\subseteq X$ homeomorphic via a map *h* to the unit interval [0, 1], such that every $x_i$ lies in $Y$. The coordinates of *x* thus inherit a total order via the total order of their images under *h*. Thus for some permutation $\sigma$ of $\{1,\ldots ,n\}$, the inherited total order has the form $x_{\sigma (1)} \le \ldots \le x_{\sigma (n)}$. We refer to $\sigma$ as the *h*-*permutation of x*. Notice that $\sigma$ only depends on the orientation determined by *h*.

#### Lemma 30

Let $x = (x_1,\ldots ,x_n) \in \textrm{Conf}(X,T)$,  $y = (y_1,\ldots , y_n)\in \textrm{Conf}_n(X)$, $Y\subseteq X$, and $h:Y\rightarrow [0,1]$ be a homeomorphism such that $x_i,y_i \in Y$ for all $1\le i\le n$. Then there is a path from *x* to *y* in $\textrm{Conf}(X,T)$ if *x* and *y* have the same *h*-permutation.

#### Proof

Let *h* and $h^{-1}$ induce maps on $\textrm{Conf}_n(Y)$ and $\textrm{Conf}_n([0,1])$ by acting component-wise. Consider the path in $\textrm{Conf}_n(X)$ from *x* to *y*

$$\begin{aligned} \gamma (t) := h^{-1}\big [(1-t)h(x) + th(y)\big ] \end{aligned}$$

whose image under *h* linearly interpolates between *h*(*x*) and *h*(*y*). Thus the *h*-permutation of $\gamma (t)$ is the same as that of *x* for all $t\in [0,1]$. To show that $\gamma$ is a path in $\textrm{Conf}(X,T)$, suppose $\textrm{Conv}_{\gamma (t)}(v)\cap \textrm{Conv}_{\gamma (t)}(v')$ is nonempty for vertices *v* and $v'$ of *T*. Then there exists *i*, *j*, and *k* such that $l_i$ and $l_k$ are descendants of $v$ (or alternatively $v'$), $l_j$ is a descendant of $v'$ ($v$), and $\gamma (t)_i\le \gamma (t)_j\le \gamma (t)_k$. Without loss of generality suppose we are in the first case. Thus $x_i\le x_j \le x_k$, since the *h*-permutation is constant along $\gamma$. So $\textrm{Conv}_x(v)\cap \textrm{Conv}_x(v')$ is also nonempty and $v\preceq v'$ or $v' \preceq v$. Hence $\gamma$ is a path in $\textrm{Conf}(X,T)$. $\square$

#### Lemma 31

Suppose $x\in \textrm{Conf}(X,T)$ consists of points on a subset $Y$ of *X* homeomorphic to the unit interval via a map *h*. Then there exists $1\le i<k \le n$ such that $l_{\sigma (i)}, \ldots , l_{\sigma (k)}$ are the descendants of $v$.

#### Proof

It suffices to show that the set $\{j \mid l_{\sigma (j)}\preceq v\}$ is convex. Consider two leaves $l_{\sigma (i)}$ and $l_{\sigma (k)}$, with $i<k$, that have $v$ as ancestor, and let $j\in \{i,\cdots , k\}$. Since $x_{\sigma (i)}<x_{\sigma (j)}<x_{\sigma (k)}$, we have $x_{\sigma (j)}\in \textrm{Conv}(x_{\sigma (i)},x_{\sigma (k)})\subseteq \textrm{Conv}_x(v)$. Since $\textrm{Conv}_x(l_{\sigma (j)}) = \{x_{\sigma (j)}\}$, $\textrm{Conv}_x(l_{\sigma (j)})$ intersects $\textrm{Conv}_x(v)$. Therefore either $l_{\sigma (j)} \preceq v$ or $v\preceq l_{\sigma (j)}$. But $l_{\sigma (j)}$ is a leaf so it must be the case that $l_{\sigma (j)} \preceq v$. $\square$

Suppose a configuration $x\in \textrm{Conf}(X,T)$ consists of points on an edge *e*, and *h* is a homeomorphism from *e* to the unit interval. Lemma 31 implies that *h* determines, for each internal node $v\in T$, its left child $Lv$ and right child $Rv$. Explicitly, we can choose $Lv$ and $Rv$ to be such that $i<j$ whenever $l_{\sigma (i)}\preceq Lv$ and $l_{\sigma (j)} \preceq Rv$. Therefore, the configuration *x* induces the structure $T_x$ of a *chiral merge tree* on $T$, as defined in (Curry 2018, Definition 5.3):

#### Definition 32

A *chiral merge tree* is a binary cellular merge tree where the two children of any internal node are labelled as either the *left* or *right* child^2^.

Since $x\in \textrm{Conf}(X,T)$ gives rise to a notion of left and right children of the internal nodes of *T*, we let $T_x$ be the chiral merge tree structure on *T* induced by *x*. The next lemma will let us alter the chiral merge tree structure assigned to a configuration, when $X$ is especially simple.

Recall that we are still proving Theorem 26, and so *T* denotes a generic cellular merge tree.

#### Lemma 33

Let $X$ be a geometric starlike tree of degree 3, i.e. $X$ is homeomorphic to three copies of [0, 1] identified at 0. Let $x\in \textrm{Conf}(X,T)$ be a configuration lying on the interior of an edge *e* of *X*. Fix a homeomorphism *h* of *e* with [0, 1]. Let $T_c$ be any chiral merge tree structure on $T$. Then there exists a path from *x* to some $y\in \textrm{Conf}(X,T)$ lying on the interior of *e*, such that $T_y=T_c$.

#### Proof

By induction, it is sufficient to prove this lemma in the case where $T_c$ differs from $T_x$ by only inverting the left child $Lv$ and right child $Rv$ of a given node $v$. Note that as cellular merge trees, $T_x$ and $T_c$ (and hence also $T_y$) are identical to $T$.

For simplicity, assume that the permutation $\sigma$ induced by *x* and *h* is the identity, thus $x_1 \le \ldots \le x_n$. Denote by $l_{i},\ldots ,l_{k}$ the descendants of $v$. There is some $i\le j < k$ such that $l_{i},\ldots ,l_{j}$ are the descendants of $Lv$ while $l_{j+1},\ldots ,l_{k}$ are the descendants of $Rv$. The goal is thus to build a path from *x* to some $y = (y_1,\ldots ,y_n)$ in $\textrm{Conf}(X,T)$, where each entry of *y* is interior to *e*, satisfying

$$\begin{aligned} y_{1} \le \ldots \le y_{i-1} \le y_{j+1} \le \ldots \le y_{k} \le y_{i}\le \ldots \le y_{j} \le y_{k+1} \le \ldots \le y_{n}. \end{aligned}$$

Let $e_1=e$, $e_2$, and $e_3$ be the edges of *X*, and *b* be the branch point of *X*. Without loss of generality assume that *h* is such that $h(b) = 0$. We will show that the following sequence of moves from *x* to *y* are allowable in $\textrm{Conf}(X,T)$:

1. Move the $1^{\text {st}}$ through $j^{\text {th}}$ coordinates from $e_1$ into $e_2$, in that order.
2. Move the $(j+1)^{\text {th}}$ through $k^{\text {th}}$ coordinates from $e_1$ into $e_3$, in that order.
3. Move the $i^{\text {th}}$ through $j^{\text {th}}$ coordinates from $e_2$ into $e_1$, in reverse order.
4. Move the $(j+1)^{\text {th}}$ through $k^{\text {th}}$ coordinates from $e_3$ into $e_1$, in reverse order.
5. Move the $1^{\text {st}}$ through $i^{\text {th}}$ coordinates from $e_2$ into $e_1$, in reverse order.

**Fig. 6:**
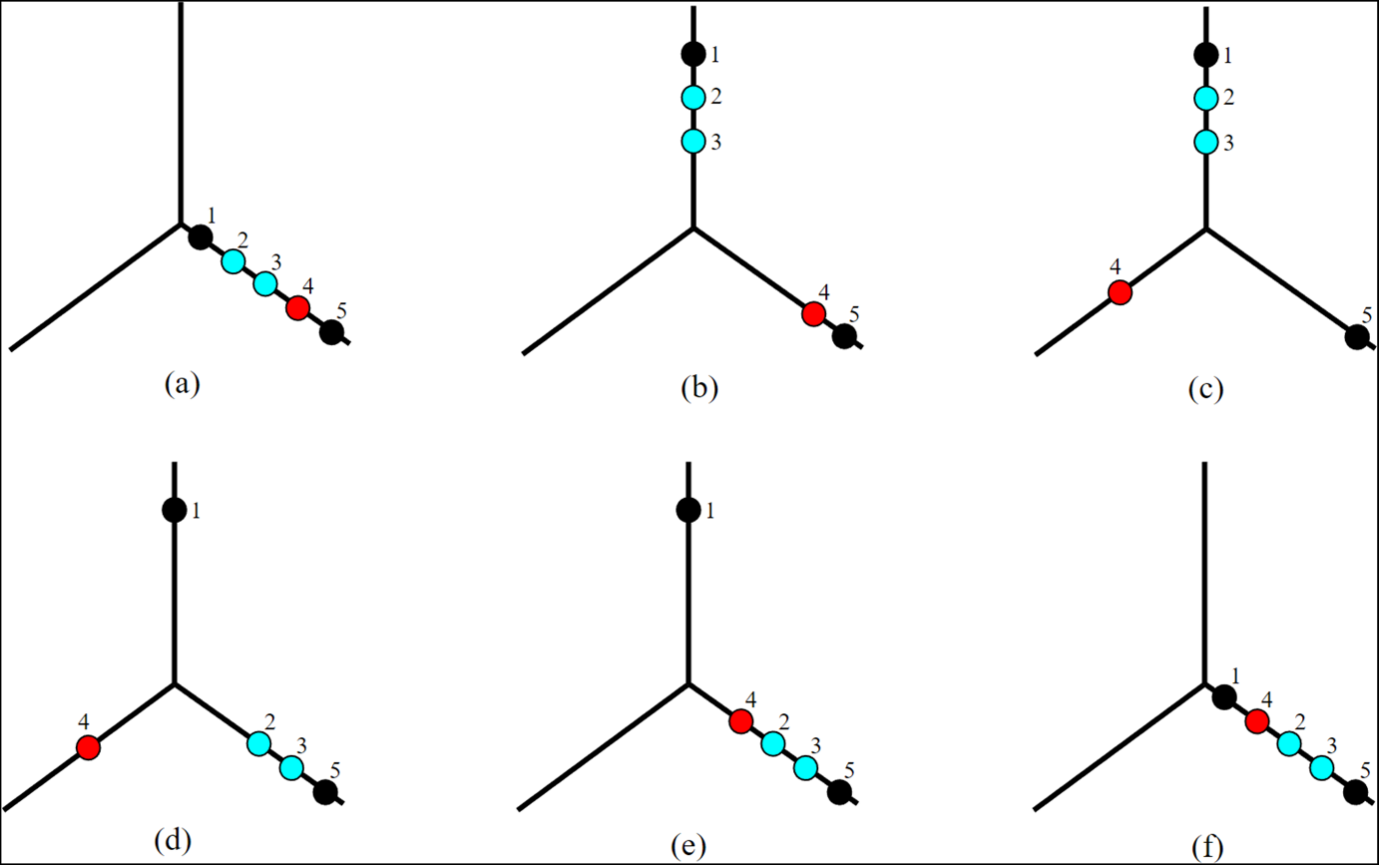
A visual representation of the proof of Lemma 33. In the depicted example, $T$ has five leaves, three of which are descendant from $v$. One of these nodes, highlighted in red, is moreover a descendant of $Lv$. The other two descendants of $v$, highlighted in blue, are descendants of $Rv$. Panels (a) through (f) show the path used to reconfigure points in the proof (colour figure online)

We remark here that this strategy of proof resembles that of Section 7 of (Mischaikow and Weibel 2021) (see also their Figure 7.1). Figure 6 shows a visualisation of this sequence of moves in an example where there are five coordinates.

Moves 1 and 5 can be realised by a path in $\textrm{Conf}(X,T)$ via Lemma 30 with $Y= e_1 \cup e_2$. For move 2, suppose we have already moved coordinates $j+1$ through $m-1$ into $e_3$ to attain a configuration $z = (z_1,\ldots , z_n)$. Let *P* denote the shortest path from $z_m$ to *b* and $z' = (z_1,\ldots ,z_{m-1}, b,z_{m+1}, \ldots , z_n)$. Let $v_0$ be a node of $T$ not ancestral to $l_m$.

Suppose that $\textrm{Conv}_z(v_0)$ intersects *P*. Since $l_m \npreceq v_0$, $\textrm{Conv}_z(v_0)$ can only intersect *P* at *b*. This means that $\textrm{Conv}_{z'}(v_0)$ intersects the interiors of both $e_2$ and $e_3$, so there must be points on $e_3$ in *z* already, i.e. $m>j+1$. In particular, $\textrm{Conv}_z(v_0)$ contains both $z_j$ and $z_{m-1}$. Since $l_j$ and $l_{m-1}$ are descendants of $Lv$ and $Rv$ respectively, $v_0$ must be an ancestor of $v$. Since $l_m$ is a descendant of $v$, $v_0$ is an ancestor of $l_m$, a contradiction. Hence $\textrm{Conv}_z(v)$ does not intersect intersect *P*, and so by Lemma 27 we can move the $m^{\textrm{th}}$ coordinate of *z* to *b*. Then applying Lemma 28 we can move the $m^{\textrm{th}}$ coordinate of *z* into the interior of $e_3$. Induction on *m* then allows us to complete move 2.

The cases of moves 3 and 4 are handled similarly. $\square$

#### Lemma 34

Let *X* be a geometric starlike tree of degree 3. Then $\textrm{Conf}(X,T)$ is path-connected.

#### Proof

Fix an edge $e\in X$ with an orientation *h*. By Proposition 23, $\textrm{Conf}(X,T)\ne \emptyset$. Let $x,y\in \textrm{Conf}(X,T)$. Up to applying Lemma 29 and Lemma 30, we can assume that *x* lies in the interior of *e*. Similarly we may assume for *y* lies in the interior of *e*.

By Lemma 33, we can connect *x* to a configuration $x'\in \textrm{Conf}(X,T)$ lying on *e* and such that $T_{x'}=T_{y}$. In particular $x'$ and *y* induce the same *h*-permutation, and therefore, thanks to Lemma 30, there is a path between them in $\textrm{Conf}(X,T)$. $\square$

Finally we can prove the central theorem of the section, restated below for convenience.

#### Theorem 26

Let *X* be a geometric tree not homeomorphic to the unit interval. Let $T$ be a generic cellular merge tree. Then $\textrm{Conf}(X,T)$ is path-connected.

#### Proof of Theorem 26

We choose a cellular structure on $X$ such that every vertex of $X$ is a leaf or a branch point. Let *l* be a leaf of $X$, and *e* be the edge incident to *l*. Since $X$ is connected and not homeomorphic to the unit interval, the other endpoint of *e* must be a branch point. Hence, there is a subtree $Y\subseteq X$ which is a geometric starlike tree of degree 3 containing *e*. By Proposition 23, $\textrm{Conf}(Y,T)\ne \emptyset$. Let $y\in \textrm{Conf}(Y,T)\subseteq \textrm{Conf}(X,T)$ be a fixed, target configuration on $X$.

Let $x = (x_1,\ldots ,x_n)\in \textrm{Conf}(X,T)$. Applying Lemma 29 and then Lemma 30, we find a path in $\textrm{Conf}(X,T)$ from *x* to a configuration $x'$ whose points lie in the interior of *e*. Viewing $x'$ as a configuration in $\textrm{Conf}(Y,T)\subseteq \textrm{Conf}(X,T)$, by Lemma 34 it can be joined to *y* via a path in $\textrm{Conf}(Y,T)$, which also defines a path in $\textrm{Conf}(X,T)$. $\square$

#### Corollary 35

Let *X* be a geometric tree not homeomorphic to the unit interval. Given a generic barcode *D*, there exists a constant $\delta >0$ such that the path connected components of the fiber $\textrm{PH}^{-1}(D)$ are at distance at least $\delta$ from each other. In particular, the path connected components of $\textrm{PH}^{-1}(D)$ are the connected components of $\textrm{PH}^{-1}(D)$.

#### Proof

Let $f,g:X\rightarrow \mathbb {R}$ be functions in distinct path connected components of the fiber $\textrm{PH}^{-1}(D)$. Then by Theorem 25, their merge trees $\textrm{MT}(f)$ and $\textrm{MT}(g)$ are non-isomorphic, and therefore by Proposition 15, we have that $\Vert f-g\Vert _\infty \ge \delta$. $\square$

#### Corollary 36

Let *X* be a geometric tree not homeomorphic to the unit interval. The fiber $\textrm{PH}^{-1}(D)$ has a finite number of connected components given by:

$$\begin{aligned}\# \pi _0(\textrm{PH}^{-1}(D))=\prod _{\begin{array}{c} [b,d)\in D\\ d \ne \infty \end{array}} \# \big \{ [b',d') \in D \mid [b,d) \subset [b',d')\big \}.\end{aligned}$$

#### Proof

This is the number of distinct cellular merge trees with barcode *D*, see (Curry 2018, Theorem 4.8). From Theorem 25, such merge trees are in bijection with path connected components of $\textrm{PH}^{-1}(D)$, which equal connected components of $\textrm{PH}^{-1}(D)$ by Corollary 36. $\square$

### Topology of connected components in the fiber

When there are very few leaves in the cellular merge tree $T$, and hence very few points in $\textrm{Conf}(X,T)$, we are able to deduce the homotopy type of the connected components of $\textrm{MT}^{-1}(T)$, and hence $\textrm{PH}^{-1}(D)$ for simple barcodes *D*. Recall that a cellular merge tree $(T,\pi )$ gives rise to a barcode $\textrm{PH}(\pi )$. We first study the simplest case, where $T$ has only one leaf.

#### Corollary 37

Let *X* be the geometric realisation of a tree, $T$ be a cellular merge tree with one leaf and *D* be the barcode associated to $T$. Then $\textrm{MT}^{-1}(T) = \textrm{PH}^{-1}(D)$ and both are contractible.

#### Proof

The barcode *D* consists of one infinite interval $[a,\infty )$, where *a* is the value assigned to the one leaf of $T$. The only merge tree that can give rise to *D* is $T$, by (Curry 2018, Theorem 4.8). Hence $\textrm{MT}^{-1}(T) = \textrm{PH}^{-1}(D)$. We can choose a cellular structure on *T* with exactly one vertex, the leaf of *T*. Recalling Definition 19, since there is only one vertex in *T*, the condition

$$\begin{aligned}\forall \text { nodes } v,v'\in T, \;\textrm{Conv}_{x}( v) \cap \textrm{Conv}_{x}( v') \ne \emptyset \Rightarrow v\preceq v' \text { or } v'\preceq v\end{aligned}$$

defining $\textrm{Conf}(X,T)$ as a subset of $\textrm{Conf}_1(X)$ is trivially true ($v$ has to be $v'$). Hence $\textrm{Conf}(X,T)=\textrm{Conf}_1(X)$. Thus by Theorem 20,

$$\begin{aligned} \textrm{MT}^{-1}(T) \simeq \textrm{Conf}(X,T) = \textrm{Conf}_1(X) = X, \end{aligned}$$

and *X* is contractible, so $\textrm{MT}^{-1}(T)$ is contractible. $\square$

If there are only two points in $\textrm{Conf}(X,T)$, its structure is still fairly simple, and has already been computed up to homotopy. We derive the following as an immediate consequence.

#### Corollary 38

Let *X* be the geometric realisation of a tree with at least one vertex of degree $\ge 3$ and let $T$ be a cellular merge tree with exactly two leaves $l_1$ and $l_2$. Suppose $\pi (l_1) \ne \pi (l_2)$. Then $\textrm{MT}^{-1}(T)$ is homotopy equivalent to the wedge sum of

$$\begin{aligned} -1 + \sum _{v\in N(X)} (\eta (v) - 1)(\eta (v) - 2) \end{aligned}$$

circles, where *N*(*X*) denotes the nodes in any cellular decomposition of *X* and $\eta (v)$ denotes the degree of node *v* in *X*. Moreover, denoting by *D* the barcode arising from the cellular merge tree $T$, we have $\textrm{PH}^{-1}(D)= \textrm{MT}^{-1}(T)$.

#### Proof

Theorem 20 tells us that $\textrm{MT}^{-1}(T)$ is homotopy equivalent to $\textrm{Conf}(X,T)$.

We can choose a cellular structure on *T* with three vertices, two of them being the leaves $l_1$ and $l_2$ and the remaining vertex being a branch point which we will denote by *w*. The condition

$$\begin{aligned}\forall \text { nodes } v,v'\in T, \;\textrm{Conv}_{x}( v) \cap \textrm{Conv}_{x}( v') \ne \emptyset \Rightarrow v\preceq v' \text { or } v'\preceq v\end{aligned}$$

defining $\textrm{Conf}(X,T)$ always holds when either *v* or $v'$ is the branch point *w*, since $v,v'\preceq w$. Without loss of generality the only remaining possibility is that $v = l_1$ and $v' = l_2$. For all $x = (x_1,x_2) \in \textrm{Conf}_2(X)$, $\textrm{Conv}_x(l_1) = x_1$ and $\textrm{Conv}_x(l_2) = x_2$. Therefore $\textrm{Conv}_{x}(l_1) \cap \textrm{Conv}_{x}(l_2) = \emptyset$ and so $x\in \textrm{Conf}(X,T)$. Hence $\textrm{Conf}_2(X) \subseteq \textrm{Conf}(X,T)$. The reverse inclusion is trivial from the definition of $\textrm{Conf}(X,T)$ and so $\textrm{Conf}_2(X) = \textrm{Conf}(X,T)$. In summary,

$$\begin{aligned} \textrm{MT}^{-1}(T) \simeq \textrm{Conf}(X,T) = \textrm{Conf}_2(X), \end{aligned}$$

and the homotopy type of $\textrm{Conf}_2(X)$ is computed in in (Farber 2018, Theorem 11.1).

The last statement follows from (Curry 2018, Theorem 4.8): *D* is the barcode with two intervals, one finite contained by the other, infinite interval, and therefore $T$ is the only merge tree giving rise to the barcode *D*. $\square$

#### Remark 3

Let *X* be the star-like tree made of *n* edges joined at one vertex, and let *D* be a barcode as in Corollary 38. Then the corollary tells us that $\textrm{PH}^{-1}(D)$ is homotopy equivalent to a wedge of $n^2 -3n +1$ circles.

In (Mischaikow and Weibel 2021), the authors consider the same barcode *D* and any discrete tree *Y* obtained from *X* by inserting at least one additional vertex in the interior of each edge. Consider the space of functions *f* on the vertices and edges of *Y*, where $f(e) = \max (f(v),f(w))$ for any edge $e = (v,w)$. In this scenario, the authors find that $\textrm{PH}^{-1}(D)$ is also homotopy equivalent to a wedge of $n^2-3n+1$ circles (Mischaikow and Weibel 2021, Theorem 8.1). This suggests that there may be a general relationship between the fiber of persistent homology of geometric trees and discrete trees with sufficiently fine triangulations.

## Data Availability

Not applicable.

## Footnotes

1 In (Munch and Stefanou 2019), labelled merge trees are instead referred to as *phylogenetic trees*.

2 Technically, in (Curry 2018) a chiral merge tree is defined as a cellular merge tree where each branch point has two children, with a fixed order on the leaves. However, as explained in (Curry 2018, Definition 5.3) this amounts to a choice of left and right edges attaching branch points *v* to their children. Therefore Curry’s definition amounts to a choice of left and right children for each branch point.

## References

[CR1] Bendich, P., Marron, J.S., Miller, E., et al.: Persistent homology analysis of brain artery trees. The annals of applied statistics **10**(1), 198 (2016) 27642379 10.1214/15-AOAS886 PMC5026243

[CR2] Catanzaro, M.J., Curry, J.M., Fasy, B.T., et al.: Moduli spaces of morse functions for persistence. Journal of Applied and Computational Topology **4**(3), 353–385 (2020)

[CR3] Chen, C., Ni, X., Bai, Q., et al.: A topological regularizer for classifiers via persistent homology. In: The 22nd International Conference on Artificial Intelligence and Statistics, pp 2573–2582 (2019)

[CR4] Cohen-Steiner, D., Edelsbrunner, H., Harer, J.: Stability of persistence diagrams. Discrete & Computational Geometry **37**(1), 103–120 (2007)

[CR5] Crawley-Boevey, W.: Decomposition of pointwise finite-dimensional persistence modules. Journal of Algebra and its Applications **14**(05), 1550066 (2015)

[CR6] Curry, J.: The fiber of the persistence map for functions on the interval. Journal of Applied and Computational Topology **2**(3), 301–321 (2018)

[CR7] Curry, J., DeSha, J., Garin, A., et al.: From trees to barcodes and back again ii: Combinatorial and probabilistic aspects of a topological inverse problem. arXiv preprint arXiv:2107.11212 (2021)

[CR8] Cyranka, J., Mischaikow, K., Weibel, C.: Contractibility of a persistence map preimage. Journal of Applied and Computational Topology **4**(4), 509–523 (2020) 33094152 10.1007/s41468-020-00059-7 PMC7548282

[CR9] Farber, M.: Configuration spaces and robot motion planning algorithms. In: Combinatorial And Toric Homotopy: Introductory Lectures, pp. 263–303. World Scientific, Singapore (2018)

[CR10] Gasparovic, E., Munch, E., Oudot, S., et al.: Intrinsic interleaving distance for merge trees. arXiv preprint arXiv:1908.00063 (2022)

[CR11] Hiraoka, Y., Nakamura, T., Hirata, A., et al.: Hierarchical structures of amorphous solids characterized by persistent homology. Proc. Natl. Acad. Sci. **113**(26), 7035–7040 (2016) 27298351 10.1073/pnas.1520877113 PMC4932931

[CR12] Johnson, B., Scoville, N.A.: Merge trees in discrete morse theory. Research in the Mathematical Sciences **9**, 1–17 (2022)

[CR13] Kanari, L., Dłotko, P., Scolamiero, M., et al.: A topological representation of branching neuronal morphologies. Neuroinformatics **16**(1), 3–13 (2018) 28975511 10.1007/s12021-017-9341-1 PMC5797226

[CR14] Kanari, L., Ramaswamy, S., Shi, Y., et al.: Objective morphological classification of neocortical pyramidal cells. Cereb. Cortex **29**(4), 1719–1735 (2019) 30715238 10.1093/cercor/bhy339 PMC6418396

[CR15] Kanari, L., Garin, A., Hess, K.: From trees to barcodes and back again: theoretical and statistical perspectives. Algorithms **13**(12), 335 (2020)

[CR16] Leygonie, J., Beers, D.: Fiber of persistent homology on morse functions. Journal of Applied and Computational Topology 1–14 (2022) 10.1007/s41468-025-00213-z PMC12414068 40922982

[CR17] Leygonie, J., Henselman-Petrusek, G.: Algorithmic reconstruction of the fiber of persistent homology on cell complexes. arXiv preprint arXiv:2110.14676 (2021)

[CR18] Leygonie, J., Tillmann, U.: The fiber of persistent homology for simplicial complexes. j. of Pure and Appl. n.a. **226**(12), 107099 (2022)

[CR19] Li, C., Ovsjanikov, M., Chazal, F.: Persistence-based structural recognition. In: Proceedings of the IEEE Conference on Computer Vision and Pattern Recognition, pp 1995–2002 (2014)

[CR20] Liu, Y., Scoville, N.A.: The realization problem for discrete morse functions on trees. In: Algebra Colloquium, World Scientific, pp 455–468 (2020)

[CR21] Mischaikow, K., Weibel, C.: Persistent homology with non-contractible preimages. arXiv preprint arXiv:2105.08130 (2021)

[CR22] Morozov, D., Beketayev, K., Weber, G.: Interleaving distance between merge trees. Discret. Comput. Geom. **49**(22–45), 52 (2013)

[CR23] Munch, E., Stefanou, A.: The -cophenetic metric for phylogenetic trees as an interleaving distance. In: Research in Data Science. Springer, p 109–127 (2019)

[CR24] Smith, P., Kurlin, V.: Families of point sets with identical 1d persistence. arXiv preprint arXiv:2202.00577 (2022)

[CR25] Touli, E.F., Wang, Y.: Fpt-algorithms for computing gromov-hausdorff and interleaving distances between trees. arXiv preprint arXiv:1811.02425 (2018)

